# Adoptive cell transfer therapy with ex vivo primed peripheral lymphocytes in combination with anti-PDL1 therapy effectively inhibits triple-negative breast cancer growth and metastasis

**DOI:** 10.1186/s12943-023-01914-8

**Published:** 2024-01-06

**Authors:** Odd L. Gammelgaard, Mikkel G. Terp, Alexei F. Kirkin, Simone Johansen, Sofie Traynor, Henriette Vever, Per Guldberg, Annette R. Kodahl, Morten F. Gjerstorff, Henrik J. Ditzel

**Affiliations:** 1 Department of Cancer and Inflammation Research, Institute of Molecular Medicine, University of Southern Denmark, J.B. Winsløws Vej 25, 5000 Odense C, Denmark; 2 Cytovac A/S, 2970 Hørsholm, Denmark; 3 Danish Cancer Institute (DCI), Copenhagen, Denmark; 4 Department of Oncology, Odense University Hospital, Odense, Denmark; 5 Department of Clinical Research, University of Southern Denmark, Odense, Denmark; 6 Academy of Geriatric Cancer Research (AgeCare), Odense University Hospital, Odense, Denmark

**Keywords:** Cellular cancer therapy, Adoptive cell transfer therapy, ALECSAT, Anti-PDL1, Triple-negative breast cancer

## Abstract

**Background:**

Adoptive cell transfer cancer immunotherapy holds promise for treating disseminated disease, yet generating sufficient numbers of lymphocytes with anti-cancer activity against diverse specificities remains a major challenge. We recently developed a novel procedure (ALECSAT) for selecting, expanding and maturating polyclonal lymphocytes from peripheral blood with the capacity to target malignant cells.

**Methods:**

Immunodeficient mice were challenged with triple-negative breast cancer cell lines or patient-derived xenografts (PDX) and treated with allogeneic or autologous ALECSAT cells with and without anti-PDL1 therapy to assess the capacity of ALECSAT cells to inhibit primary tumor growth and metastasis.

**Results:**

ALECSAT mono therapy inhibited metastasis, but did not inhibit primary tumor growth or prolong survival of tumor-bearing mice. In contrast, combined ALECSAT and anti-PDL1 therapy significantly inhibited primary tumor growth, nearly completely blocked metastasis, and prolonged survival of tumor-bearing mice.

**Conclusions:**

Combined ALECSAT and anti-PDL1 therapy results in favorable anti-cancer responses in both cell line-derived xenograft and autologous PDX models of advanced triple-negative breast cancer.

**Supplementary Information:**

The online version contains supplementary material available at 10.1186/s12943-023-01914-8.

## Background

Adoptive cell transfer (ACT) therapy using either chimeric antigen receptor (CAR) T cells or ex vivo expanded tumor infiltrating lymphocytes (TILs) has shown immense potential in oncology [1–4], but the application of either strategy as a common cancer therapy remains challenging. CAR T cell therapy is limited by its monospecific reactivity, which promotes the outgrowth of antigen-negative variants and additionally carries the risk of on-target off-cancer toxicity. Consequently, this therapy is primarily used for treatment of hematologic cancers [5], although recent data also demonstrates promising potential in the context of solid cancers [6]. TIL cultures can be generated from most cancers provided the tumor tissue is resectable and contains lymphocytes. However, melanoma is currently the only cancer type that consistently gives rise to cancer-recognizing TIL cultures [7]. Hence, accelerating the development of cellular therapies to leverage their potency and benefit more patients is highly desirable. This is particularly critical in cancers with few therapeutic opportunities such as metastatic triple-negative breast cancer (TNBC).

ALECSAT is a novel type of ACT therapy generated from blood by generation of proliferating antigen-presenting CD4^+^ T helper cells and exposing these cells to demethylating agents to induce expression and presentation of methylation-silenced gene products, such as cancer germ-line antigens. The modified antigen-presenting cells are subsequently used to selectively expand lymphocytes that respond to these antigens [8]. Preparation of ALECSAT can be accomplished through minimally invasive intervention without restrictions regarding tumor location or lymphocyte content. ALECSAT was recently evaluated in patients with late-stage and newly diagnosed glioblastoma, respectively [8, 9] and was well tolerated. A subset of patients displayed tumor regression and accumulation of ALECSAT cells at the relevant tumor sites, although overall survival was not improved.

Here, we evaluated the anti-cancer effect of ALECSAT therapy in vitro and in vivo using a modified production method for generation of ALECSAT cells called ALECSAT II (A_II_). Recapitulating previous clinical trials, we found suboptimal activity of ALECSAT as monotherapy. Importantly, the addition of anti-PDL1 greatly augmented anti-cancer activity translating into inhibited tumor growth, metastatic control, and prolonged survival in cancer cell line- and patient-derived xenograft (PDX) models of TNBC. The PDX models were established with tumor and immune cells from the same patients to confirm that the observed benefit was not an allogenic effect. This combined treatment strategy should be generally applicable for most cancers.

## Methods

### Cancer cell culture

The human TNBC cell lines MDA-MB-231 and MDA-MB-468 were obtained from American Type Culture collection (ATCC). Cells were grown in Dulbecco’s modified Eagle medium (DMEM) AQmedia (Sigma-Aldrich, D0819), supplemented with 10% fetal bovine serum (FBS) and 1% Penicillin–Streptomycin (P/S).

### Clinical samples

As part of a phase Ib clinical trial examining A_II_ in combination with carboplatin and gemcitabine metastasis biopsies from three patients with TNBC were collected from 2019 to 2022 at the Odense University Hospital with informed consent. TNBC diagnoses were made by a trained histopathologist. This study was carried out according to the principles of the Helsinki Declaration and approved by the National Ethical Committee of Denmark (no. S-1906975).

### Flow cytometry

Cells were blocked by human Fc block (BD, 564220) and stained with combinations of Pacific blue CD3 (SK7, Biolegend), FITC CD56 (TULY56, eBioscience), Alexa Fluor 647 CD56 (NCAM, Biolegend), PE CD8 (SK1, Biolegend), FITC CD4 (SK3, Biolegend), FITC CD45RA (HI100, Immunotools), FITC CD45RO (UCHL1, Immunotools), FITC mouse IgG2a (PPV-04, Immunotools), Alexa Fluor 647 CD62L (DREG-56, Biolegend) Alexa Fluor 647 CD28 (CD28.2, Biolegend), Alexa Fluor 647 CD27 (M-T271, Biolegend), Alexa fluor CCR7 (150503, BD), Alexa Fluor 647 CD107a (H4A3, Biolegend), Alexa Fluor 647 PD1 (MIH4, BD), or Alexa Fluor 647 PDL1 (2340D, R&D systems). Cells incubated with anti-HLA-A2 antibodies (551230, BD) were subsequently stained with an Alexa Fluor 488-labeled goat anti-human IgG (Life Technologies, A11013). Damaged cells were excluded by TO-PRO-3 staining (ThermoFischer, T3605) or LIVE/DEAD™ Fixable Near-IR Dead Cell Stain Kit (ThermoFischer, L10119). Data were analyzed using FlowJo software (version 10.8.0).

### Lentiviral transduction

The generation of Luciferase 2 (Luc2)-expressing MDA-MB-231 cells has been previously described [10]. MDA-MB-468 cells were stably transduced by lentiviral transduction. Briefly, the Luc2 expression plasmid (Addgene 75020) was prepared as lentivirus by calcium phosphate co-transfection HEK293T cells together with the packaging plasmids (pHIT60 and pCOltGaIV). Virus was harvested from the supernatant after 3 days, filtered, precipitated with polyethylene glycol (PEG) and resuspended in PBS. Cancer cells (5 × 10^4^) were supplemented with lentivirus and 5 mg/mL polybren overnight and 72 h after infection, stably transduced cells were sorted based on expression of mCherry. HLA-A2^+^ MDA-MB-468 cells were generated similar to the above described Luc2 transduction but instead using the pMP71-HLA-0201-His vector (Addgene, 108214). Sorting was based on binding of the anti-HLA-A2 antibody.

### Co-culture studies

Cancer cells (5 × 10^3^) were suspended in AIM V medium (Gibco, 12055–083) supplemented with 2% human serum and seeded in a white 96-well plate and allowed to attach for 2 h. Immune effector cells were subsequently added to the wells and incubated for 24 h at 37 °C. After incubation, cancer- cell viability was assessed by addition of D-luciferin (3 mg/ml in PBS) and luminescence was immediately measured using a Victor3 Multilabel Plate Reader. In some assays additional blockers were added, including anti-PD1 (pembrolizumab) and anti-PDL1 (atezolizumab). Cancer-cell viability was calculated as: Viability = (sample – background)/(Cancer cells only – background) × 100%

### Isolation of immune cells

CD3^+^ and CD56^+^ cells were purified using the Dynabeads Untouched Human T cells (Invitrogen, 11344D) and the EasySep Human CD56 Positive Selection Kit II (Stem cell, 17,855), according to manufacturer’s instructions, respectively.

### Degranulation assays

Cancer cells (1 × 10^5^) were suspended in growth medium (AIM V medium supplemented with 5% human serum). Effector cells (3 × 10^5^) were subsequently added together with growth medium supplemented with 2 μg/mL, GolgiStop (BD, 554724), or growth medium supplemented with GolgiStop and anti-CD107a. Upon 5 h incubation cells were harvested, stained and analyzed by flow cytometry.

### In vivo experiments

All animal experiments were performed at the animal core facility at the University of Southern Denmark. Mice were housed under pathogen-free conditions with ad libitum food and water. The light/dark cycle was 12 h light/dark, with light turned on from 6 a.m. to 6 p.m. Housing temperature was 21 ± 1 °C and relative humidity 40–60%. Sample size was guided by previous experiments and pre-liminary data. No animals were excluded from analysis. If not stated otherwise, no randomization was performed as treatment was given before tumor size could be reliably determined. Investigators performing the experiments were not blinded. Mice were acclimatized for 2 weeks before initiation of experiments. A schematic outline of animal experiments was created using Biorender.com.

#### Generation of patient-derived xenograft (PDX) models

Female NOG (NOD.Cg-Prkdc^SCID^Il2*rg*^*tm1Sug*^/JicTac, Taconic) mice were anesthetized and the fourth mammary fat pad was surgically exposed and injected with 50 μL of extracellular matrix (ECM) gel (Merck, E1270-5). The mammary fat pad was subsequently opened, and a tumor piece (approximately 8 mm^3^) was implanted in the ECM gel. The mammary fat pad and skin were subsequently closed by internal and external stitches, respectively.

#### Comparison between injection routes

Female NOG (*n* = 5) or hIL15 NOG (NOD.Cg-*Prkdc*^*scid*^ *Il2rg*^*tm1Sug*^ Tg(CMV-IL2/IL15)1-1Jic/JicTac, Taconic) (*n* = 3) were injected with 1 × 10^6^ MDA-MB-231 cells into the fourth mammary fatpad. Three days later, 10^7^ A_II_ cells were injected intravenously. In parallel, separate mice were surgically transplanted with MDA-MB-231 tumor pieces into the mammary fat pat using the ECM gel (Merck, E1270-5) with (*n* = 3) or without (*n* = 3) A_II_ cells.

#### Primary tumor growth, spontaneous metastasis and survival

Female NOG mice were transplanted with fresh MDA-MB-231 tumor pieces in ECM gel with or without 5 × 10^6^ A_II_ cells or injected 1 × 10^6^ MDA-MB-231 cells in the mammary fat pad. Alternatively, female hIL15 NOG were transplanted with a PDX tumor piece in ECM. Autologous A_II_ cells (5 × 10^6^) were subsequently injected intravenously. Anti-PDL1 (200 μg Atezolizumab) were administered intraperitoneally on day 0, 3 and weekly until day 100 or termination of the experiment, whichever came first. Mice were sacrificed as tumors reached 1.2 cm in diameter.

#### Experimental metastasis

Female hIL15 NOG mice were injected intravenously with 1 × 10^6^ MDA-MB-231 cells. Seven days later mice were injected with 5 × 10^6^ A_II_ cells. Anti-PDL1 (200 μg atezolizumab) were administered intraperitoneally on day 0 and 3 and weekly until termination.

### Tumor dissociation

Tumors were harvested for dissociation into single-cell suspensions using the Tumor Dissociation Kit, mouse (Miltenyi Biotec, 130–095-730) according to the manufacturer’s description. Briefly, tumors were cut in small pieces of approximately 2 mm in diameter and mixed with the dissociation cocktail and subsequently placed in the gentleMACS Dissociator. Upon dissociation, cell suspensions were filtered using a 70 μm cell strainer (BD, # 352350), washed in DPBS and resuspended in 2 mL of red blood cell lysing buffer (155 mm NH4Cl, 12 mm NaCO3, and 0.1 mm EDTA) and gently mixed for 1 min at room temperature. Following 2 × wash in complete DPBS, the single cells were counted and stained for flow cytometry.

### RNA sequencing

RNA was purified using RiboZol (VWR) or TRI Reagent (Sigma-Aldrich) as previously described [11]. For tissues, this step included homogenization using 2.8 mm zirconium oxide beads (Precellus) and a Precellus 24 homogenizer (3 × 15 s, 6500 rpm). Purified RNA was prepared for sequencing on the Illumina NovaSeq 6000 Sequencing Platform using the NEBNext Poly(A) mRNA Magnetic Isolation Module (New England Biolabs, Herlev, Denmark, E7490L) and the NEBNext Ultra II DNA Library Prep Kit for Illumina (New England Biolabs, E7645L) with unique dual indexes according to the manufacturer’s instructions. The quality of raw sequencing reads was assessed using FASTQC (Babraham Bioinformatics, Braham Institute, Cambridge, Great Britain), and adaptor sequences were removed using the FASTX toolkit. Trimmed and filtered sequencing reads were aligned to the human (hg38) and mouse (mm10) genomes using Spliced Transcripts Alignment to a Reference (STAR) software with default parameters [12]. Tags in exons were counted using iRNA-seq [13]. Transcripts per million were averaged in each treatment group and gene-set enrichment analysis, GSEA 4.3.2, software (Cambridge, MA, USA) was used to identify the enriched gene sets in the group treated with A_II_ and A_II_ in combination anti-PDL1.

### T cell receptor sequencing

T cell receptor (TCR) sequencing was performed using the SMARTer Human TCR a/b Profiling kit (Takara 635016) according to the manufacturer’s protocols. Libraries for both alpha- and beta-chain diversity were generated in the same experiment using 1 µg of total RNA as starting material. The TCR libraries were sequenced on an Illumina NovaSeq 6000 platform using read lengths of 150 bp read 1, 8 bp i7 index, 150 bp read 2, 8 bp i5 index and 20% PhiX. Pre-processing of sequencing reads, UMI-based analysis, clonotype calling and statistical analysis was performed using the Cogent NGS Immune Profiler Software v1.0 (Takara). Output from the Cogent Profiler was further processed in R.

### Immunohistochemistry (IHC)

Tissue sections from the formalin-fixed and paraffin-embedded tissue blocks were cut (3 µm). IHC-staining was performed on DAKO OMNIS from Agilent or BenchMark Ultra from ROCHE. Sections were initially deparaffinized and rehydrated prior to antigen retrieval by boiling in either Cell Conditioning 1 buffer (Ventana Medical Systems, 05279801001) for 48 min at 100 °C (PDL1, Ventana Medical Systems, SP142), 32 min at 100 °C (CD3, Roche Diagnostics, 2GV6), 15 min microwave (TEG buffer, DAKO), 32 min at 100 °C (CD8, Agilent Technologies, M710301-2, C8/144B (1:100)), 32 min at 100 °C (pan-cytokeratin, Agilent Technologies, M351501-2, 1:100)) or target retrieval solution (Agilent Technologies, S236784-2) for 40 min at 97 °C (PDL1, Agilent Technologies, 22C3). Sections were incubated with primary antibody for 16 min at 36 °C (PDL1, SP142), 40 min at 32 °C (PDL1, 22C3), 8 min at 36 °C (CD3), 60 min at room temperature (CD11b), 32 min at 36 °C (CD8), or 24 min at 36 °C (pan-cytokeratin). Primary antibody binding was detected with either OptiView DAB IHC detection kit (760–700; Ventana Medical systems; PDL1 (SP142), CD3, CD11b, CD8, pan-cytokeratin) or Envision FLEX DAB (Agilent Technologies; PDL1 (22C3)) as chromogen. To prepare double stainings of Ki67 and CD3, antigen retrieval was achieved by boiling in Cell Conditioning 1 buffer for 48 min at 100 °C. Sections were incubated with primary anti-Ki67 antibody (Roche Diagnostics, 30–9) for 12 min at 36 °C and detected by Optiview DAB IHC. Sections were subsequently stained with anti-CD3 (Roche Diagnostics, 2GV6) for 12 min at 36 °C and detected by ultraView Universal. All sections were counterstained with hematoxylin. Hematoxylin and eosin staining was performed on a DAKO Coverstainer (Agilent). Slides were scanned using a NANOZOOMER 2.0-HT Whole Slide Imager (Hamamatsu, San Diego, CA, USA). PDL1 immune cell positivity was scored by a trained pathologist based on the Ventana PD-L1 (SP142) assay.

### Quantification of metastases

The NDP.view 2.3.14 software (Hamamatsu) annotation tool was used to markup full section and tumor area in lung and liver respectively. Metastatic load was calculated as tumor area / tissue area × 100%. Lesions/mm^2^ was calculated as number of metastases / tissue area.

### Quantification of immunohistochemical stained sections

The ImageJ software 1.53a was used to compare CD45, CD3 and CD11b density as well as PDL1 staining intensity in primary tumors, livers, spleens, and lung metastases using the adjust color threshold function.

### Generation of ALECSAT I (AI) and ALECSAT II (AII)

A_I_ cells were prepared as previously described [8]. A_II_ cells were prepared similar to A_I_ cells, but with the addition of autologous dendritic cells at day 15 of culture. The rational for the addition of dendritic cells was provided by previously published data showing that dendritic cells can improve the process of immunization by providing “tonic” signals required for subsequent antigen stimulation [14, 15]. This modification resulted in a significant increase of the total number of generated cells (up to 8–tenfold) without changes in the principal characteristics of cells such as expression of the differentiation markers CD62L, CD27 and CCR7 (Fig. S1). The A_II_ procedure consists of four steps: (1) generation of mature dendritic cells; (2) co-culture of mature dendritic cells with lymphocytes with addition of IL2 leading to intensive proliferation of predominantly CD4^+^ cells; (3) treatment of activated lymphocytes with the DNA demethylating agent 5-Aza-2’-deoxycytidine (5-aza-CdR) leading to induction of the expression of variety of cancer germline antigens, and (4) co-culture of purified lymphocytes with the 5-aza-CdR-treated activated lymphocytes and fresh dendritic cells (immunization step). The employed cultivation medium for generation of dendritic cells consisted of serum-free AIM-V medium with addition of 2 mM L-glutamine. Cultivation of lymphocytes was performed in the same medium with addition of 2% autologous plasma-derived serum. Generation of dendritic cells was performed by culturing monocytes in the presence of GM-CSF and IL4 for 4 days with subsequent culturing of cells in the presence of IL1β (10 ng/ml), TNF-α (10 ng/ml), IL6 (1000 IU/ml) and prostaglandin E2 (0.2 µg/ml) for 2 days. Mature dendritic cells were cocultured with thawed lymphocytes for 7 days (days 6–13) with addition of 25 IU/ml of IL2. After 7 days of coculture, lymphocytes were harvested and cultured for the additional 2 days (days 13–15) in the presence of 150 IU/ml of IL2 and 10 µm of 5-aza-CdR. Thereafter, 5-aza-CdR-treated cells were washed and co-cultured with a new portion of intact lymphocytes and dendritic cells (ratio 10:10:1). IL2 and fresh medium were added at days 17, 20, 22 and 24. At day 26, cells were harvested and used for the experiments.

### Statistical analysis

Data were analyzed using GraphPad Prism v.8 software and are represented as mean ± SEM or mean ± SD of independent biological replicates. Statistical analyses were performed as described in the figures. Differences were considered significant based on P values (*, *P* < 0.05; **, *P* < 0.01; ***, *P* < 0.001).

## Results

### AII contains cancer-eradicating T cells with unique phenotypes

**Fig. 1:**
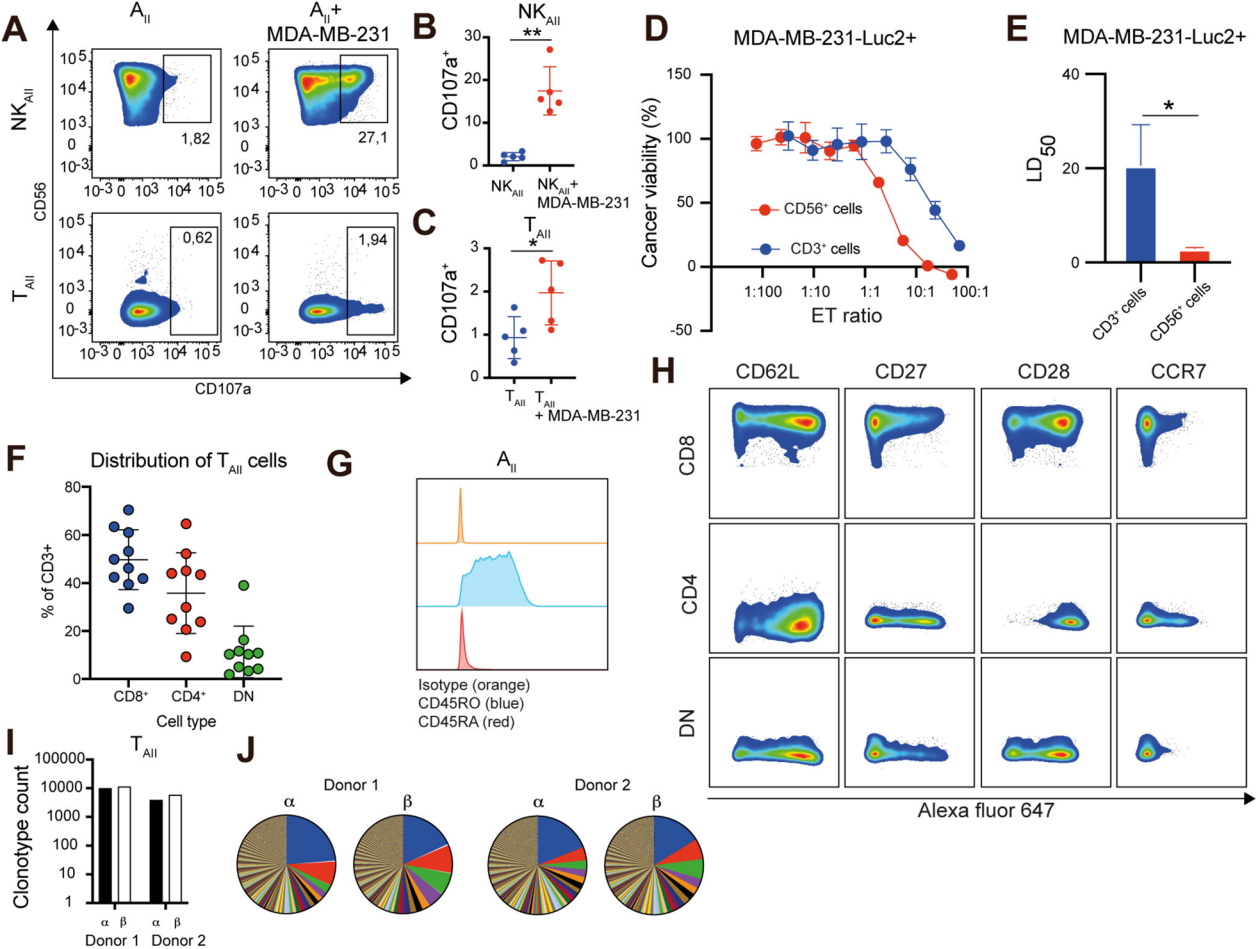
A_II_ consists of phenotypically novel T cells with cancer-eradicating capacity. **A** Degranulation analysis of A_II_ upon 5 h culture alone, or with MDA-MB-231 cells, demonstrating degranulation of both NK_AII_ and T_AII_ cells. A representative of five experiments is shown. **B**-**C** Quantification of the degranulation of NK_AII_ and T_AII_ upon co-culture with MDA-MB-231 cells from five independent experiments presented as mean ± SD. **D** Cancer cell viability analysis (luminescence) following 24-h co-culturing with purified immune cell fractions. A representative of seven independent experiments is shown. Data is presented as mean ± SEM of triplicates. **E** Comparison of the LD_50_ from the data presented in **D**. **F** Comparison of percentage of CD4^+^, CD8^+^, and DN T_AII_ cells in 10 A_II_ preparations evaluated by flow cytometry. **G** Flow cytometry analysis of A_II_ cells showing expression of CD45RO, but not CD45RA. **H** Phenotypic analysis of CD4^+^, CD8.^+^ and DN T_AII_ cells with regard to CD62L, CD27, CD28 and CCR7 expression determined by flow cytometry **I**-**J** TCR clonotype analysis identifying thousands of TCR alpha and beta chains in each A_II_ preparation. Preparations are dominated by 10–20 T cell clones. Statistical difference was determined using the paired t-test **B**-**C** and the Student’s t-test **E**. *0.05 > *P* ≥ 0.01, **0.01 > *P* ≥ 0.001

The dose of tumor-reactive immune cells positively correlated with outcome in both mice and humans [16–18]. We therefore aimed at modifying the original ALECSAT (A_I_) expansion protocol to increase cell numbers. Addition of dendritic cells at the beginning of the immunization step (day 15) significantly increased the expansion efficiency without qualitative differences in terms of cell content and expression of immunological markers relevant for adoptive cancer immunotherapy (Fig. S1a-b, *P* < 0.001). The highly expanded cell product (referred to as A_II_) is composed of a mixture of natural killer (NK) and T cells (NK_AII_ and T_AII_ cells), both of which possess the ability to recognize and kill MDA-MB-231 TNBC cells in vitro (Fig. 1a-e and Fig. S1d-e). Analysis of individual A_II_ preparations generated from 10 healthy donors revealed comparable levels of CD4^+^ and CD8^+^ cells as well as a smaller population of CD4^−^CD8^−^ (DN) cells (Fig. 1f). To further characterize the phenotype of the various cell types, the expression of CD45RO, CD45RA, CCR7, CD27, CD28 and CD62L was analyzed by flow cytometry. The majority of A_II_ cells were CD45RO^+^CD45RA^−^, indicating that they had become activated during culture and acquired a central memory (CM, CD62L^+^CCR7^+^CD27^+^) or effector memory (EM, CD62L^−^CCR7^−^CD27^±^) phenotype (Fig. 1g and Fig. S1f) [19]. Similar to A_I_ cells, the majority of CD4^+^, CD8^+^, and DN T_AII_ cells were CD62L^+^CCR7^−^CD27^±^CD28^±^ (Fig. 1h), indicating that they represent a phenotypically, and possibly functionally, novel type of T cells. To determine the width of the TCR repertoire of T_AII_, we performed a TCR clonotype analysis of A_II_ preparations from two healthy donors. Each preparation contained thousands of TCR alpha and beta chains, with 10–20 clones accounting for approximately 50% of the cells (Fig. 1i-j). Taken together, our studies demonstrate that T_AII_ cells exhibit a unique phenotype and are capable of recognizing cancer cells in vitro. An unexpected width of the TCR repertoire of T_AII_ cells was further observed.

### The delivery route of AII cells greatly influences in vivo survival and expansion

**Fig. 2:**
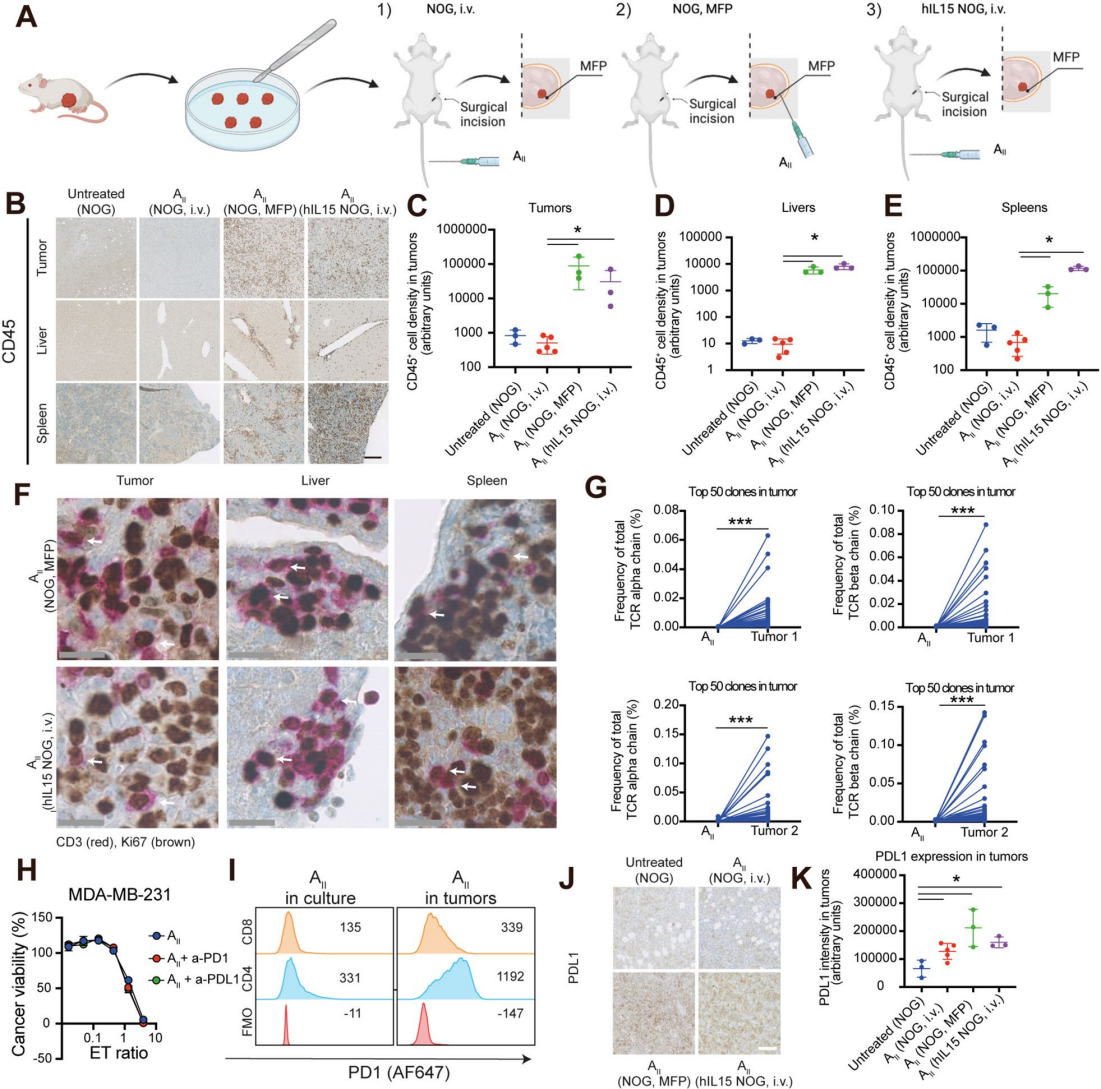
Injection route dictates anti-cancer activity of A_II_ therapy. **A** Schematic outline of the three investigated conditions of animal experiments. **B** On day 27 tumors and organs were excised and analyzed by IHC. Panels show representative images of tumors, livers and spleens stained for CD45, demonstrating higher CD45 levels in all tissues for both hIL15 NOG, i.v. and NOG, MFP conditions compared to NOG, i.v. Primary tumor expansion for this animal experiment is shown in Fig. S3. *n* = 5 (NOG, untreated), *n* = 5 (NOG, i.v.), *n* = 3 (NOG, MFP), *n* = 3 (hIL15 NOG, i.v.) **C**-**E** Quantification of the immune cell density (CD45^+^) in tumors, livers and spleens, respectively. **F** Same as in **B**, but co-stained for CD3 (red) and Ki67 (brown). White arrows indicate proliferating (double positive) cells. **G** TCR alpha and beta chain analysis comparing the frequencies of the 50 most prevalent chains in tumors with their frequency prior to administration, showing selective expansion of these clones. **H** Cancer killing assay as in Fig. 1d showing no added benefit of blocking PD1 or PDL1 (10 μg/mL). **I** Flow cytometry analysis of the PD1 level of CD4^+^ and CD8^+^ A_II_ cells in culture and in tumors, demonstrating increased PD1 expression in tumors. Numbers indicate geometric mean fluorescence intensity. **J** Same as in **B** but stained for PDL1 showing increased PDL1 expression in tumors for both hIL15 NOG, i.v. and NOG, MFP conditions. **K** Quantification of PDL1 tumor expression. Statistical difference was determined by the Mann Whitney test **C**, **D**, **E** or the paired t-test **G** or Student’s t-test **K**. *0.05 > *P* ≥ 0.01, ***0.001 > *P.* Black, white and grey scale bar 250, 100 and 25 μm, respectively. i.v.; intravenous

We utilized the human TNBC model MDA-MB-231 to evaluate the in vivo anti-cancer activity of A_II_ cells. This model was selected based on the following parameters: 1) robust tumor growth, 2) capacity to metastasize, 3) expression of the HLA-A2 allele, and 4) in vitro recognition by both NK_AII_ and T_AII_ cells. Despite the effective anti-cancer killing by A_II_ cells observed in vitro, inhibition of tumor growth was undetectable in vivo when up to 10^7^ A_II_ cells were administrated intravenously, and autopsy revealed almost complete absence of tumor-infiltrating A_II_ cells (Fig. S2). Others have demonstrated that in vivo vaccination and common gamma-chain cytokine support greatly enhance the activity of adoptively transferred T cells [16, 20]. To improve the tumor homing and survival of A_II_ cells we, in parallel, 1) transplanted MDA-MB-231 tumor pieces into the mammary fat pad (MFP) and injected A_II_ cells intravenously in NOG mice (control), 2) co-transplanted MDA-MB-231 tumor pieces with A_II_ cells directly into the MFP (to enhance antigen-specific stimulation), or 3) transplanted MDA-MB-231 tumor pieces into the MFP and injected A_II_ cells intravenously in NOG mice, which produce human IL15 (hIL15 NOG) to enhance common gamma chain support (Fig. 2a). The latter two strategies significantly enhanced the number of tumor-infiltrating A_II_ cells compared to intravenous administration in NOG mice, where the number of tumor-infiltrating CD45^+^ cells remained low (Fig. 2b-c, *P*< 0.05). Importantly, both strategies also enhanced A_II_ homing to distant organs such as spleen, liver and lungs, suggesting that A_II_ cells exerted full body immune surveillance (Fig. 2b-e, *P* < 0.05). In vivo expansion of T_AII_ was indicated by co-staining IHC analysis showing proliferating (Ki67^+^) CD3^+^ cells in both liver, spleen and tumor (Fig. 2f) and confirmed by TCR clonotyping analysis of the injected A_II_ cells and tumors (Fig. 2g, *P* < 0.001). There was also a tendency towards smaller tumor size in the two latter groups, but the difference did not reach statistical significance (Fig. S3a-b). The paradoxical co-existence of A_II_ cells, with the capacity to kill cancer cells, and viable cancer cells led us to hypothesize that the lack of response could be attributed to adaptive resistance mechanisms enforced by the tumors. Although blocking PD1 or PDL1 by itself did not affect the killing capacity in vitro (Fig. 2h), we noted that PD1 expression on CD4^+^ and CD8^+^ T_AII_ cells increased following tumor infiltration (Fig. 2i). Furthermore, PDL1 expression was higher in A_II_-treated tumors compared to untreated tumors (Fig. 2j-k, *P* < 0.05). Taken together, these data demonstrate that the survival of T_AII_ cells is greatly enhanced by injection directly into the area of MFP surrounding the tumor or by hIL15 stimulation. Furthermore, our data indicate that T_AII_ cells are functional in vivo and stimulate tumor PDL1 expression.

### Combined AII and anti-PDL1 therapy inhibits TNBC primary tumor growth

**Fig. 3:**
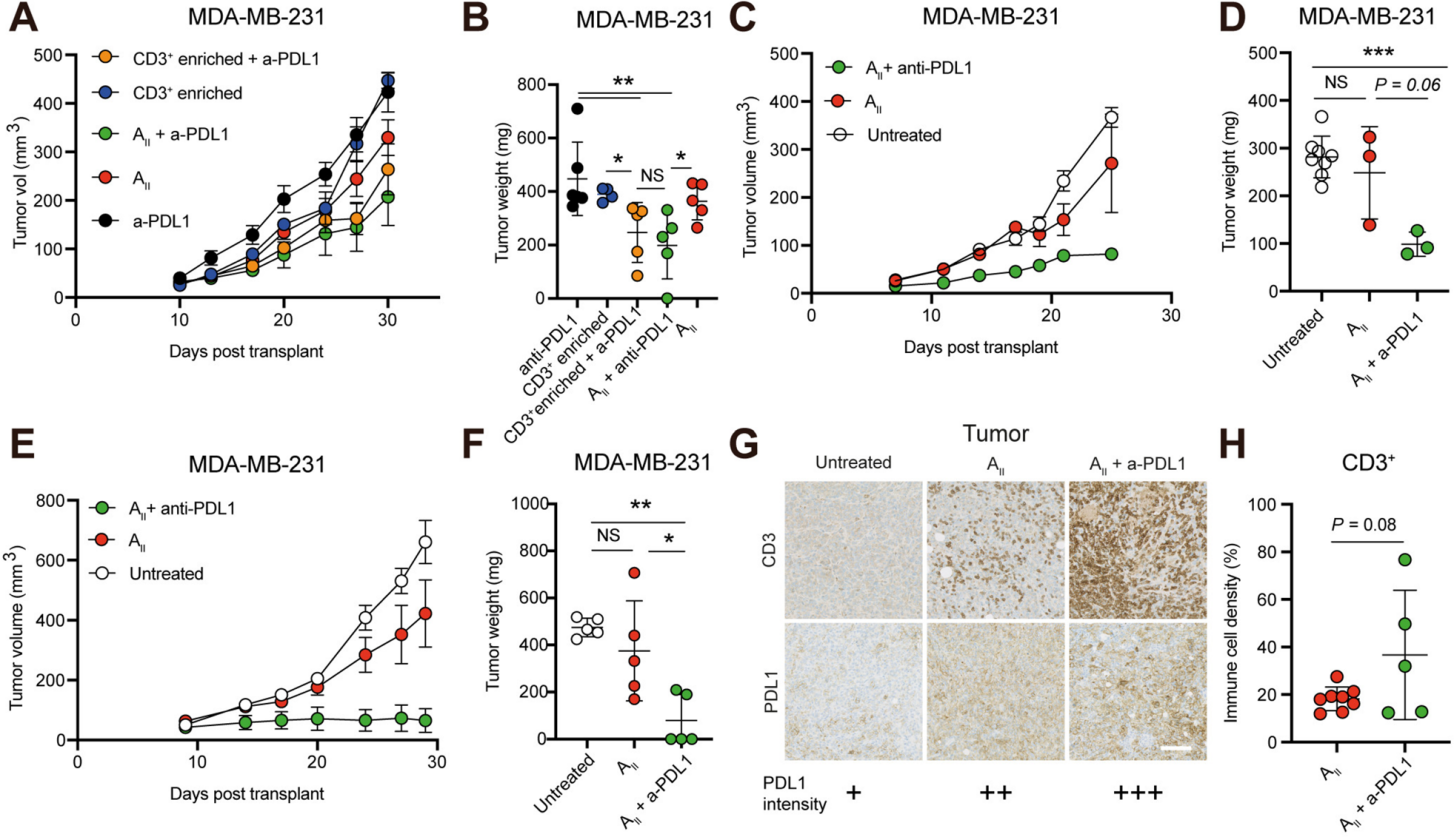
Anti-PDL1 blockade enhances the therapeutic efficacy of A_II_ therapy. **A** Growth of orthotopically transplanted MDA-MB-231 tumors in female NOG mice treated with either anti-PDL1 (*n* = 6), A_II_ (*n* = 5), A_II_ in combination with anti-PDL1 (*n* = 5), CD3^+^ enriched A_II_ cells (*n* = 4), or CD3^+^ enriched A_II_ cells in combination with anti-PDL1 (*n* = 5). Tumor size is presented as mean ± SEM.** B** Excised MDA-MB-231 tumors from (A) with tumor masses presented as mean ± SD, demonstrating that combined A_II_ and anti-PDL1 exert anti-cancer activity, which is retained in the CD3^+^ enriched fraction. **C**-**F** As in **A**-**B** but using two other HLA-A2 + donors, demonstrating anti-cancer activity of combined A_II_ and anti-PDL1 (donor 2: untreated (*n* = 8), A_II_ (*n* = 3), and A_II_ + anti-PDL1 (*n* = 3). Donor 3: untreated (*n* = 5), A_II_ (*n* = 5), and A_II_ + anti-PDL1 (*n* = 5). **G** Upon tumor excision tumors were analyzed by IHC. Panels show representative images of tumors stained for CD3 and PDL1 demonstrating tumor infiltration of CD3^+^ cells in tumors treated with A_II_ and A_II_ in combination with anti-PDL1 as well as increased tumor PDL1 expression. **H** Quantification of the density of CD3^+^ cells in tumors from **C**-**F**. In all experiments mice were administered 200 µg anti-PDL1 i.p. on day 0 and 3, followed by a weekly injection until termination. Statistical difference was determined by the Mann Whitney test **B**, **F** or Student’s t-test **D**, **H**, *0.05 > *P* ≥ 0.01, **0.01 > *P* ≥ 0.001, ***0.001 > *P.* NS, non-significant; a-PDL1, anti-PDL1. White scale bar 100 μm

The enhanced cancer expression of PDL1 in A_II_-treated tumors (Fig. 2j-k) prompted us to investigate whether blocking the PD1/PDL1 axis would support the therapeutic activity of A_II_. For this purpose, we used the PDL1 targeting antibody atezolizumab (Tecentriq), which is EMA-approved for PDL1-positive advanced TNBC [21, 22]. Since atezolizumab monotherapy does not exert anti-cancer activity in NOG mice (Fig. S4), we only included an anti-PDL1-treated or -untreated group in each experiment. MDA-MB-231 tumor pieces were embedded with or without A_II_ cells from an HLA-A2^+^ donor into the MFP of NOG mice and treated weekly with anti-PDL1 starting on the day of tumor transplantation. To assess the potential contribution of T_AII_ cells and NK_AII_ cells we included groups with purified CD3^+^ A_II_ cells with and without anti-PDL1 therapy. Tumors treated with either anti-PDL1, A_II_ and CD3^+^ enriched A_II_ cells as monotherapies expanded at similar pace (Fig. 3a-b). In contrast, anti-PDL1 in combination with CD3^+^ enriched cells or A_II_ demonstrated significant tumor growth inhibition (Fig. 3a-b, *P*< 0.01). There was no significant difference in the effect of combined A_II_ and anti-PDL1 compared to combined CD3^+^ enriched A_II_ cells and anti-PDL1, demonstrating that the effector population is within the CD3^+^ population (i.e. not NK_AII_ cells). To investigate the robustness of these data we repeated the analysis using another two HLA-A2^+^ donors. As anticipated, A_II_ as monotherapy did not exert significant anti-cancer activity on primary tumor growth, while anti-PDL1 in combination with A_II_ demonstrated strong tumor growth inhibition (Fig. 3c-f, *P*< 0.01). Subsequent IHC analysis of the tumors that were not completely eliminated confirmed infiltration of CD3^+^ cells in the groups receiving either A_II_ or A_II_ and anti-PDL1 as well as increased PDL1 tumor expression (Fig. 3g-h). To examine the changes induced by adding anti-PDL1 to the A_II_ therapy, we compared RNA expression levels of tumors treated with A_II_ and A_II_ in combination with anti-PDL1. As expected, tumors treated with the combination exhibited increased expression of pathways associated with T cell responses such as IL2-STAT5 signaling and IFNγ and TNFα responses (Fig. S5a). As expected, genes associated with Th1, but not Th2 or Th17, responses, were markedly increased (Fig. S5b). Taken together, these data demonstrate that treatment with combined A_II_ and anti-PDL1 exerts anti-cancer activity on primary tumors by a T_AII_ cell-dependent mechanism.

### Suppression of metastasis formation by AII therapy is potentiated by anti-PDL1

**Fig. 4:**
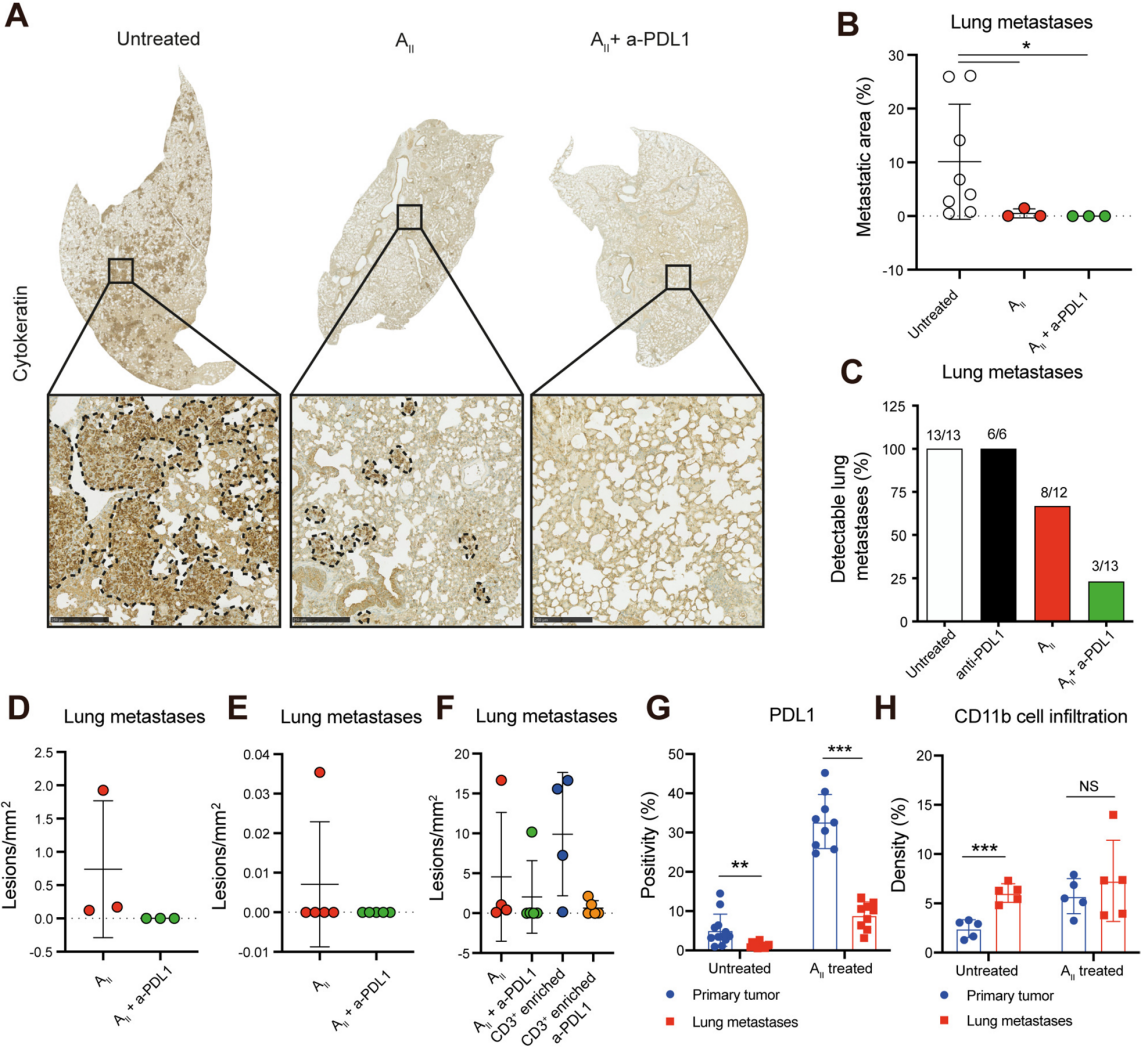
Spontaneous metastasis formation is suppressed by A_II_ therapy. **A** Representative IHC panels of lungs stained for pan-cytokeratin from NOG mice transplanted with MDA-MB-231 tumor pieces and left untreated, treated with A_II_ or A_II_ in combination with anti-PDL1 demonstrating less cancer tissue in lungs treated with A_II_ and A_II_ in combination with anti-PDL1. Bottom pictures are enlarged versions of the black insert. Dotted lines represent tumor borders. Primary tumor expansion for this animal experiment is shown in Fig. 3c. **B** Quantification of spontaneous lung metastases from **A** presented as mean ± SD. **C** Quantification of mice presenting with lung metastases. Mice from three experiments are pooled. Primary tumor expansion for these animal experiments is shown in Fig. 3a, c and e. **D**-**F** Quantification of the density of treatment-resistant lung lesions in mice treated with A_II_ or A_II_ in combination with anti-PDL1. Primary tumor growth for these animal experiments is shown in Fig. 3a, c and e). Quantification of PDL1 levels **G** and myeloid tumor cell infiltration **H** on untreated and A_II_ treated primary tumors and matched lung lesions demonstrating higher PDL1 expression in primary tumors than in lung metastases, but comparable levels of CD11b^+^ cells upon treatment. Statistical difference was determined by the Mann Whitney test **A** or Student’s t-test **G** and **H**, respectively *0.05 > *P* ≥ 0.01, **0.01 > *P* ≥ 0.001, ***0.001 > *P.* a-PDL1, anti-PDL1. Scale bar 250 μm

We previously demonstrated that MDA-MB-231 cells develop spontaneous lung and liver, but not brain metastases in the presence of allogeneic human leukocytes [23, 24]. Since A_II_ cells appeared to perform full body immune surveillance (Fig. 2b-e), we investigated whether the therapeutic effect shown in Fig. 3 for primary tumors also affected the formation of spontaneous metastasis to the lungs. As expected, untreated mice and mice treated with anti-PDL1 presented with extensive lung metastases (Fig. 4a-b and Fig. S3). Remarkably, A_II_ as monotherapy and in combination with anti-PDL1 significantly inhibited lung metastases (Fig. 4a-b and Fig. S6, *P* < 0.05). Indeed, across the three experiments only 23% (3/13) and 67% (8/12) of mice receiving A_II_ in combination with anti-PDL1 and as monotherapy, respectively, exhibited detectable lung metastases (Fig. 4c). In contrast, 100% of untreated mice (13/13) and 100% of anti-PDL1 treated mice (6/6) presented with lung metastases, and these were generally much larger (Fig. 4a and Fig. S6). The amount of treatment-resistant lesions tended to be smaller in the combination group than in the A_II_ monotherapy group, although it did not reach statistical significance (Fig. 4d-f). The anti-metastatic activity was retained in the CD3^+^ -enriched fraction, indicating that T cells play a crucial role in limiting metastasis (Fig. 4f and Fig. S6). Since metastases appeared more sensitive to A_II_ mediated killing compared to primary tumors, we analyzed the extent of PDL1 expression and recruitment of myeloid cells. To our surprise, but consistent with our in vivo observations, metastases expressed significantly lower amounts of PDL1 compared to primary tumors. In contrast, there were no significant differences between the extent of myeloid cell infiltration (Fig. 4g-h, *P* < 0.01). Taken together, these data demonstrate that A_II_ cells exert strong anti-metastatic activity independent of anti-PDL1 therapy. The data further suggests that the extent of PDL1 expression limits anti-cancer activity, and that anti-PDL1 augments the beneficial effect.

**Fig. 5:**
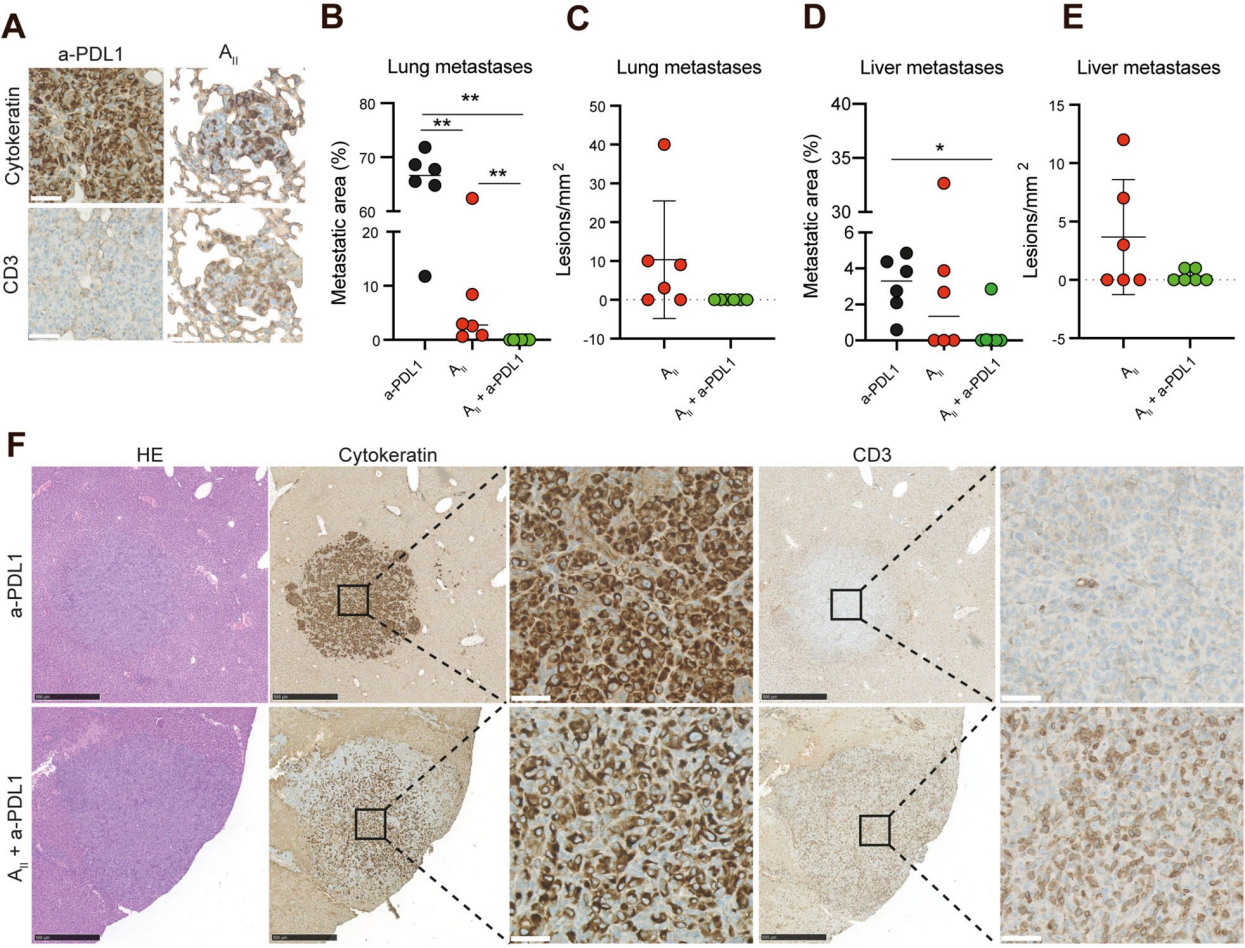
Established metastases are eradicated by A_II_ in combination with anti-PDL1. **A** Representative IHC analysis showing CD3^+^ T_AII_ homing to spontaneous lung metastases in NOG mice. **B** Quantification of lung metastases. Female hIL15 mice were challenged with an i.v. injection of 10^6^ MDA-MB-231 cells on day 0. On day 7, mice were treated with anti-PDL1 alone (*n* = 6), A_II_ alone (*n* = 6), or combined A_II_ and anti-PDL1 (*n* = 6). A_II_ and anti-PDL1 were administered i.v. and i.p., respectively. 200 µg anti-PDL1 were administered on days 7 and 10 and then once weekly. On day 29, mice were sacrificed, and organs were excised and used for IHC analysis. Mean is shown. **C** Quantification of the tumor lesions/mm^2^ of the lungs presented in B. mean ± SD is shown. **D**-**E** As in B-C, but for the tumor lesions/mm^2^ of liver sections. **F** Representative IHC analysis of size-matched tumor lesions in liver demonstrating extensive CD3 T_AII_ cell infiltration in tumors of mice treated with combined A_II_ and anti-PDL1 vs those treated with anti-PDL1 alone. Statistical difference was determined by the Mann Whitney test **B** and **D** *0.05 > *P* ≥ 0.01, **0.01 > *P* ≥ 0.001, ***0.001 > *P.* NS non-significant, a-PDL1 (anti-PDL1). Black and white scale bar 500 and 50 μm, respectively

Next, we investigated whether the anti-metastatic activity was related to limiting the spread or to actively eliminating disseminated cells. IHC analysis of lung sections suggested the latter since T_AII_ cells homed to cancerous tissue in the lung (Fig. 5a). To examine this, we evaluated the efficacy of intravenously injected A_II_ towards established experimental metastases. In these experiments, hIL15 NOG mice were challenged with an intravenous injection of MDA-MB-231 cells and 7 days later were treated by A_II_ or combined A_II_ and anti-PDL1. Similar to the observations from the spontaneous metastasis models, a strong anti-metastatic effect of A_II_ as monotherapy was observed, but addition of anti-PDL1 significantly augmented the effect (Fig. 5b, *P* < 0.01). In the excised lungs, metastases were detected in all mice receiving A_II_ monotherapy, while no single cancer cells or metastases could be detected in the lungs of mice receiving combined A_II_ and anti-PDL1 therapy (Fig. 5c). Evaluating the livers revealed multiple liver metastases in 100% (6/6) of anti-PDL1 treated mice and in 50% (3/6) of mice treated with A_II_ alone. In contrast, we detected no cancer cells in livers of 67% (4/6) of mice treated with the combination of A_II_ and anti-PDL1. The 33% (2/6) of mice presenting with detectable cancer cells only had solitary liver metastases (Fig. 5d-e). Compared to size-matched untreated liver metastases, the treatment-resistant metastases appeared less dense and extensively infiltrated by T_AII_ cells (Fig. 5f). Taken together, these data demonstrate that A_II_ cells can home to, detect and eradicate established metastases, and that the anti-metastatic effect is significantly augmented by the addition of anti-PDL1 therapy.

### The combination of AII and anti-PDL1 exerts anti-cancer activity in autologous systems

**Fig. 6:**
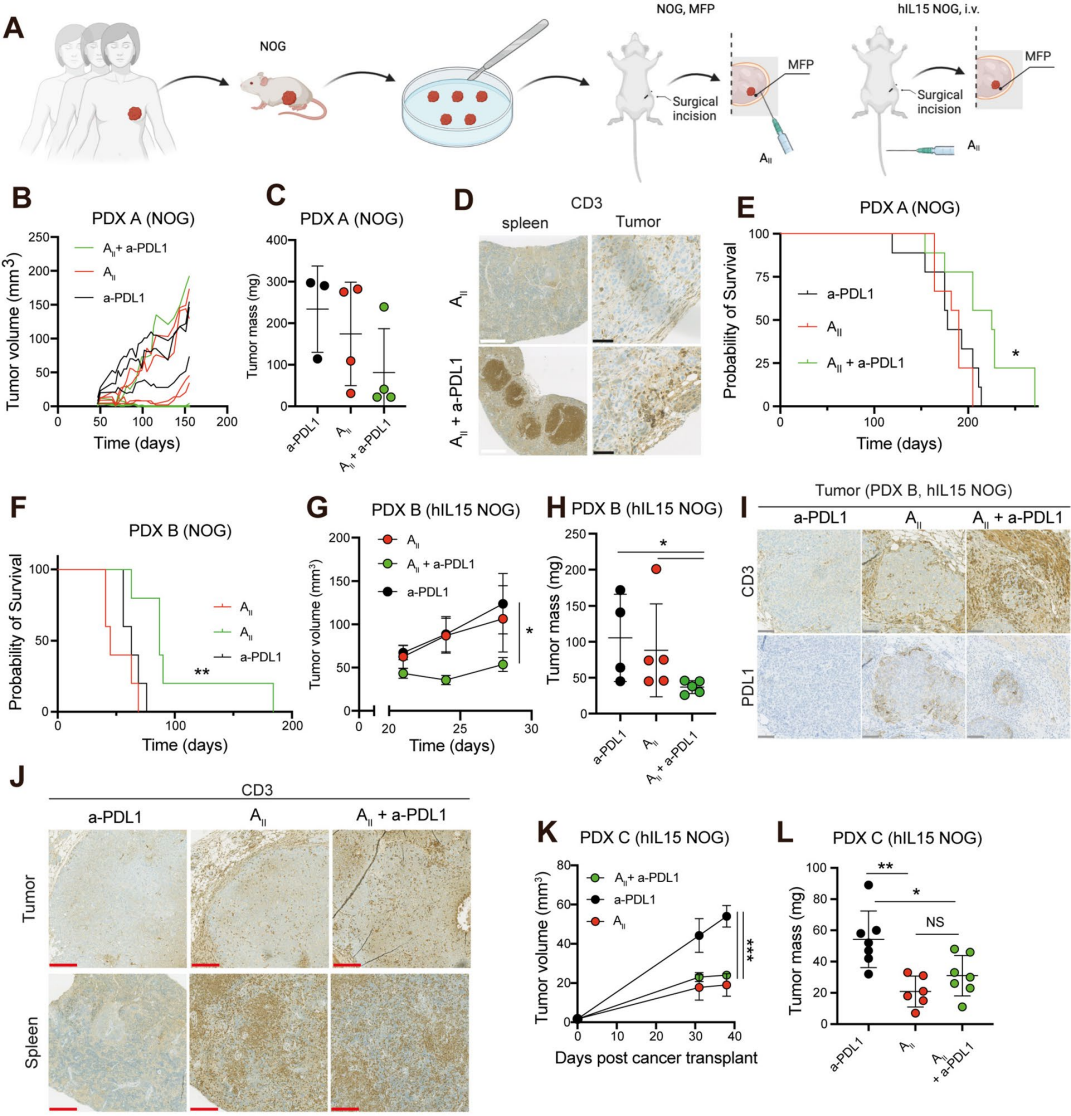
Combined A_II_ and anti-PDL1 exert enhanced anti-cancer activity in autologous PDX models. **A** Schematic outline for the generation of TNBC PDX models and evaluation of autologous A_II_ in these models. **B** Tumor growth of PDX A-derived tumors in female NOG mice treated with either anti-PDL1 alone (*n* = 3), A_II_ alone (*n* = 4) or combined A_II_ and anti-PDL1 (*n* = 4). **C** Mass of excised PDX A tumors from B. **D** IHC analysis of tumors 150 days post administration of A_II_ cells demonstrating survival of CD3^+^ T_AII_ cells in both tumor and spleen when co-treated with anti-PDL1. **E** Same PDX models as in B, but with larger groups: treated with anti-PDL1 alone (*n* = 9), A_II_ alone (*n* = 9) or combined A_II_ and anti-PDL1 (*n* = 9). **F** Tumor growth of PDX B-derived tumors in female NOG mice treated with either anti-PDL1 alone (*n* = 5), A_II_ alone (*n* = 5) or combined A_II_ and anti-PDL1 (*n* = 5). **G** Growth of PDX B in female hIL15 NOG mice when treated with anti-PDL1 alone (*n* = 5), i.v. A_II_ alone (*n* = 5) or combined i.v. A_II_ and anti-PDL1 (*n* = 5). **H** Tumor mass of excised tumors from G. **I** IHC analysis of excised tumors from G, showing enhanced PDL1 expression in CD3^+^ T_AII_ infiltrated tumors. **J** IHC analysis of excised tumors and spleen from G demonstrating T_AII_ homing to tumor and spleen. **K** Growth of PDX C in female hIL15 NOG mice when treated with anti-PDL1 alone (*n* = 7), i.v. A_II_ alone (*n* = 6) or combined i.v. A_II_ and anti-PDL1 (*n* = 7). **L** Mass of excised PDX C tumors from K. In all experiments mice were administered 200 µg anti-PDL1 i.p. on days 0 and 3 and then weekly until day 100. Tumor size and tumor mass are presented as mean ± SEM and mean ± SD, respectively. Statistical difference was determined by log-rank (Mantel-Cox) test **E** and **F**, by the two-way ANOVA method followed by following Bonferoni’s multiple correction testing **G** and **K**, or by the Mann Whitney test **I** and **L**. *0.05 > *P* ≥ 0.01, **0.01 > *P* ≥ 0.001, ***0.001 > *P.* NS non-significant. Black, white, grey, and red scale bar 50, 500, 100, and 250 μm, respectively

To rule out that the anti-cancer activity observed in Figs. 3 and 4 was a result of allogeneic rejection, we created PDX models from patients with metastatic TNBC enrolled in the A_II_ clinical trial (clinical trial gov ID: NCT04609215) and treated these with autologous A_II_ cells (Fig. 6a). First, we co-implanted PDX A (PDL1 expression on immune cells: < 1%) and autologous A_II_ cells into the MFP of NOG mice. Due to limited tumor material at the time of A_II_ generation, group sizes were limited to 3–4 mice per group. Intriguingly, 75% (3/4) mice receiving A_II_ in combination with anti-PDL1 did not display tumor outgrowth, whereas tumors in 100% (3/3) mice treated with anti-PDL1 alone and 75% (3/4) mice treated with A_II_ alone expanded (Fig. 6b-c). It is noteworthy that T_AII_ cells (CD3^+^) were detectable in both spleen and liver as late as 150 days after administration when combined with anti-PDL1 (Fig. 6d), but not when administered as monotherapy. Although encouraging, these differences were not statistically significant. Thus, we repeated the experiment with larger groups and monitored the survival of mice receiving mono or combination therapy. Combined A_II_ and anti-PDL1 conferred a statistical survival benefit compared to A_II_ alone or anti-PDL1 alone (Fig. 6e, *P*< 0.05). To confirm these findings, we established a second TNBC PDX model (PDX B, PDL1 expression on immune cells: 4%) and found a similar significant survival benefit of the combination therapy compared to either of the monotherapies (Fig. 6f, *P*< 0.01). To investigate whether intravenously injected A_II_ cells would home to autologous cancer tissue, we also evaluated the activity in hIL15 NOG mice using the PDX B model. The hIL15 NOG mice began to develop graft-versus-host like symptoms after approximately 1 month and thus had to be terminated while tumors were relatively small. Nevertheless, tumors of mice receiving the combination therapy expanded slower and were significantly smaller at endpoint compared to those treated with either monotherapy (Fig. 6g-h, *P*< 0.05). As expected, tumors of mice treated with A_II_ alone or combined A_II_ and anti-PDL1 exhibited enhanced PDL1 expression (Fig. 6i), suggesting an A_II_-mediated reactivity towards the tumor cell population. Furthermore, A_II_ cells were easily detectable in tumors and spleens demonstrating adequate tumor and lymphoid homing capacity (Fig. 6j). Finally, we generated a third TNBC PDX model (PDX C, PDL1 expression on immune cells: 2%), and evaluated the activity of autologous A_II_ cells in the hIL15 model. Consistent with the previous experiment, mice developed graft-versus-host like symptoms after approximately 1 month. Tumors treated with A_II_ or A_II_ and anti-PDL1 expanded significantly slower than anti-PDL1-treated tumors (Fig. 6k-l, *P*< 0.05). Taken together, these data demonstrate that A_II_ cells can home to cancer tissue and exert anti-cancer activity in autologous systems both when administered intravenously or directly into the MFP when combined with anti-PDL1. Further, our data indicates that even tumors with very low immune cell PDL1 expression on immune cells can benefit from combined A_II_ and anti-PDL1 therapy. Finally, it suggests that blocking PDL1-delivered signals can improve the survival of A_II_ cells.

## Discussion

Immune checkpoint inhibition therapy has shown remarkable anti-cancer activity in highly immunogenic cancers like melanoma, lung and bladder cancer. However, the effect of checkpoint inhibition is complex and only approximately 10% of cancer patients respond adequately to checkpoint therapy even though many have tumor-infiltrating T cells [25]. Thus, having the right amount of T cells with the necessary characteristics (e.g. capacity to recognize, kill, persist and evade suppression) is critical for therapeutic activity. In TNBC enhanced levels of TILs correlate with better survival [26–28]. However, the clinical benefit of blocking PD1/PDL1 in combination with chemotherapy is currently restricted to a small subset of TNBC patients [21, 22, 29], likely because the majority of patients generate insufficient numbers of tumor-reactive lymphocytes with the necessary characteristics. We previously reported that tumor-reactive lymphocytes can be generated from peripheral blood using the ALECSAT protocol [8]. Here, we extend this discovery by demonstrating how the complementary mechanisms of A_II_ and anti-PDL1 are necessary and sufficient to obtain favorable anti-cancer immunity. Using mice xenografted with TNBC cell lines or TNBC PDX models, we demonstrate that combined A_II_ and anti-PDL1 therapy limits tumor expansion, blocks metastasis and prolongs survival. Considering our findings in this study, it is recommended to also evaluate the safety and activity of combined A_II_ and anti-PDL1 therapy.

Metastases remain a major clinical challenge in oncology, accounting for more than 90% of cancer-related deaths [30]. Remarkably, our work demonstrates that A_II_ alone inhibits the development of both spontaneous and experimental metastases despite being unable to control primary tumor growth. The beneficial effect of A_II_ cells on experimental metastases demonstrates that the effect is not just a result of limiting seeding, but that the A_II_ cells are capable of locating, identifying and eliminating established metastatic lesions. We cannot rule out the possibility that metastases established over longer periods of time can create a more suppressive tumor microenvironment, thus limiting the effect of A_II_ cells, similar to what we have seen in the growth of primary tumors. To that end, it is encouraging that the anti-cancer activity of A_II_ cells towards both primary tumor growth and metastases is strongly enhanced when combined with anti-PDL1 therapy, and complete cancer eradication was seen in a small subset of mice. Nevertheless, it is likely that additional drug combinations will be necessary to achieve complete cancer eradication in a larger subset of mice.

The anti-cancer efficacy of ACT is directly related to the dose and inversely related to the differentiation state of T cells [16, 20, 31–37]. While higher and repeated doses of A_II_ cells are administered clinically, the differentiation state of A_II_ cells is currently more difficult to control. Furthermore, the observed phenotype of A_II_ does not fall within the classical definitions of naïve, stem-cell-like memory, central memory, effector memory or effector T cells, which makes it challenging to compare with the current literature. Nevertheless, we observed expression of CD27, CD28 and CD62L on many T_AII_ cells, which have been associated with clinical responses and high anti-cancer activity and persistence [20, 38–40]. Indeed, T_AII_ cells were detectable in a xenogeneic environment more than 150 days after injection without any exogenous cytokine support and without causing noticeable xenogeneic damage to the host, implying that A_II_ cells do not react against the normal tissue of the mice.

The anti-cancer activity of ACT therapy is also associated with the capacity to traffic to secondary lymphoid tissue. Indeed, LTα knockout mice, which (like NOG mice) develop disorganized white splenic pulp and lack peripheral lymphoid structures [41, 42], do not benefit from adoptive transfer of tumor-reactive central memory CD8^+^ cells, whereas WT mice do [33]. Despite the compromised lymphoid tissue in NOG mice, A_II_ cells were able to survive, expand and cause cancer growth inhibition when administered in combination with anti-PDL1. It is tempting to speculate that the beneficial effect would have been even stronger in a host with functional lymphoid structures and endogenous adaptive immunity, and without the limitations of xenogeneic trophic support [43]. The suboptimal trophic support and lack of lymphoid structures may provide an explanation for the dependency of injecting A_II_ cells directly into the MFP. We anticipate that the presence of A_II_ cells in the vicinity of cancer cells immediately stimulates the release of trophic factors such as IL2, and that the systemic concentrations either become sufficient to keep A_II_ cells alive after leaving the tumor or, more realistically, that cancer-stimulated A_II_ cells become independent or self-sufficient in providing such signals. It is well established that common gamma chain cytokine support positively impacts ACT therapy [16]. Administration of A_II_ in the vicinity of cancer cells in patients might be problematic, but our data suggests that this may in part be circumvented by co-administration of cytokines such as IL2 or IL15.

The most powerful anti-cancer responses were seen when MDA-MB-231 tumors were treated with allogeneic A_II_ cells from partially HLA-matched donors. Although a part of the response may originate from allogenicity rather than specific recognition of cancer cells, strong anti-cancer activity was also seen in all three PDX models treated with autologous (and hence HLA matched) A_II_ cells and anti-PDL1, demonstrating that allogenicity is not a requirement for cancer cell detection and destruction by A_II_. Indeed, the accumulation of T_AII_ cells in tumor tissue, upregulation of tumor PDL1, and benefit of anti-PDL1 therapy are all consistent with T_AII_ cell-mediated cancer inhibition.

We did not observe any toxicity with either A_II_ alone or in combination with anti-PDL1 in any of the NOG mice. It is important to acknowledge that xenogeneic studies may not be suitable for evaluating potential organ toxicity issues. Notably, A_I_ and A_II_ has been administered to over 151 patients with various cancer types and no signs of toxicity have been reported. Additionally, A_II_ therapy in combination with carboplatin and gemcitabine is currently undergoing evaluation in a phase Ib trial for patients with metastatic TNBC (ClinicalTrials.gov ID: NCT04609215), and thus far there have been no indications of toxicity.

Despite the Impassion130 trial failing to show survival benefits of combined atezolizumab and nab-paclitaxel in advanced TNBC regardless of PDL1 status [22], the recent findings from the Keynote355 trial have demonstrated that combined pembrolizumab and chemotherapy improves overall survival in advanced TNBC patients who have a combined positive score (CPS) of ≥ 10 [29]. We anticipate that a high CPS reflects ongoing anti-cancer responses that are necessary for obtaining the clinical benefit of blocking the PD1/PDL1 pathway [44]. Most encouragingly, our data strongly suggests that even in patients lacking sufficient anti-cancer immune responses, combined checkpoint blockade and tumor-reactive lymphocytes therapy, such as A_II_, may be an attractive therapeutic strategy.

## Conclusions

We identify combined ALECSAT and anti-PDL1 therapy as a potent treatment for achieving a favorable anti-cancer immune response in TNBC. Since anti-PDL1 is already approved and ALECSAT is under investigation for advanced TNBC, our findings have immediate translational relevance for patients with advanced TNBC.

### Supplementary Information

**Additional file 1:** **Supplementary Fig. 1.** The ALECSAT II expansion protocol generates a higher number of qualitatively comparable effector cells than the ALECSAT I expansion protocol. **A-B** Comparison of the total number of generated ALECSAT cells using either the ALECSAT I or II expansion protocols, demonstrating a significant increase using the latter. **C** Comparison of the proportion of CD4^+^ or CD8^+^ T cells as well as NK cells in generated A_I_ and A_II_ products from five different donors, showing a slight increase in CD4^+^ T cells. **D-E** Cancer cell viability analysis (luminescence) following 24-hour co-culturing with A_I_ or A_II_ cells generated in parallel from the same donors. Data is presented as mean ± SEM of triplicates. **F** Phenotypic analysis of CD8^+^ T cells with regard to CD62L (*n*=5), CD27 (*n*=5) and CCR7 (*n*=3) expression in A_I_ and A_II_ cells generated in parallel determined by flow cytometry. Statistical difference was determined by the paired t-test **B** or Student’s t-test **C** and **F**. *0.05 > *P* ≥ 0.01, ***0.001 > *P*

**Additional file 2:** **Supplementary Fig. 2.** A_II_ cells perish upon i.v. injection in NOG mice. **A** Growth of orthotopically transplanted MDA-MB-231 tumors in female NOG mice left untreated (*n*=8), treated with an i.v. injection of 10^6^ A_II_ cells (*n*=15) or 10^7^ A_II_ cells (*n*=15) on day 14. Tumor size was measured on day 13 and mice were randomized to treatment groups. A pool of two A_II_ donors is shown. Data is presented as mean ± SD. **B** On day 24, tumors from A were excised and tumor mass determined. Data is presented as mean ± SD. **C** IHC analysis of excised tumors from A and B showing lack of CD3^+^ cells. Scale bar 100 μm. Statistical difference was determined by Students t-test B.

**Additional file 3:** **Supplementary Fig. 3.** hIL15 stimulation or injection of A_II_ cells in the vicinity of tumors is insufficient to inhibit tumor growth. **A** Growth of MDA-MB-231 tumors in female NOG and hIL15 NOG mice. Cancer cells were either injected in suspension or small tumor pieces were transferred into the MFP as indicated in the figure legend. Mice challenged with MDA-MB-231 cells in suspension were either left untreated (*n*=5) or treated with an i.v. injection of 10^7^ A_II_ cells on day 3 and 9 (*n*=5, NOG and *n*=3 hIL15 NOG). Mice receiving MDA-MB-231 tumor pieces were either left untreated (*n*=3) or treated with an injection of 10^7^ A_II_ directly into the same MFP as the tumor piece on day 0 and an i.v. injection of 10^7^ A_II_ cells on day 9 (*n*=3). Tumor size is presented as mean ± SEM. **B** MDA-MB-231 tumors were excised on day 29 and tumor mass were measured and presented as mean ± SD. Statistical difference was determined by the Mann Whitney test B.

**Additional file 4:** **Supplementary Fig. 4.** Anti-PDL1 therapy is ineffective as monotherapy in NOG mice. **A** Growth of orthotopically-transplanted MDA-MB-231 tumors in female NOG mice left untreated (*n*=4) or treated with anti-PDL1 (*n*=4). (B) On day 33, tumors from A were excised and tumor mass determined. Data is presented as mean ± SD. Quantification of spontaneous lung **C** and liver **D** metastases from A presented as mean ± SD. Statistical differences were determined by the two-way ANOVA method following Bonferoni’s multiple correction testing A or the Student’s t-test B-D, respectively *0.05 > *P* ≥ 0.01, **0.01 > *P* ≥ 0.001, ***0.001 > *P*. a-PDL1, anti-PDL1.

**Additional file 5:** **Supplementary Fig. 5.** Cancer control is associated with T cell activity. **A** Enrichment plots of significantly enriched gene sets in tumors treated with A_II_ in combination with anti-PDL1 compared to those treated with A_II_ as monotherapy showing strengthened T cell responses. **B** Comparison of selected genes associated with Th1 (green), Th2 (red) and Th17 responses (yellow) showing a consistent increase in Th1-, but not Th2- or Th17-associated genes.

**Additional file 6:** **Supplementary Fig. 6.** A_II_ cells suppress spontaneous metastasis formation in NOG mice. **A** Quantification of spontaneous lung metastases from the animals presented in figure 3e presented as mean ± SD. **B** Representative IHC panels of lungs stained for pan-cytokeratin from A. Dotted lines represent tumor borders. **C-D** As in A-B with primary tumor expansion shown in figure 3a. Statistical difference was determined by the Mann Whitney A or unpaired t-test C, respectively *0.05 > *P* ≥ 0.01, **0.01 > *P* ≥ 0.001, ***0.001 > *P*. NS, non-significant; a-PDL1, anti-PDL1. Black scale bar 250.

## Data Availability

All relevant data are available within the article and supplementary files, or available from the authors upon reasonable request.

## Abbreviations

ACT: Adoptive cell transfer
ALECSAT: Autologous Lymphoid Effector Cells Specific Against Tumor
ALECSAT II: A_II_
CAR: Chimeric antigen receptor
DMEM: Dulbecco’s modified Eagle medium
ECM: Extracellular matrix
FBS: Fetal bovine serum
IHC: Immunohistochemistry
Luc2: Luciferase 2
MFP: Mammary fat pad
NK: Natural Killer
NK_AII_: ALECSAT NK
PDL1: Programmed death-ligand 1
PEG: Polyethylene glycol
PDX: Patient derived xenograft
P/S: Penicillin–Streptomycin
T_AII_: ALECSAT T
TCR: T cell receptor
Th: T helper
TILs: Tumor infiltrating lymphocytes
TNBC: Triple-negative breast cancer

## References

[1] Neelapu SS, Locke FL, Bartlett NL, Lekakis LJ, Miklos DB, Jacobson CA (2017). Axicabtagene ciloleucel CAR T-Cell therapy in refractory large B-cell lymphoma. N Engl J Med.

[2] Maude SL, Laetsch TW, Buechner J, Rives S, Boyer M, Bittencourt H (2018). Tisagenlecleucel in children and young adults with B-cell lymphoblastic leukemia. N Engl J Med.

[3] Schuster SJ, Svoboda J, Chong EA, Nasta SD, Mato AR, Anak O (2017). Chimeric antigen receptor T cells in refractory B-cell lymphomas. N Engl J Med.

[4] JBAG Haanen MR, T.H. Borch, J.H. van den Berg, Ö. Met2, M. Geukes Foppen, J. Stoltenborg Granhøj, B. Nuijen, C. Nijenhuis, J.H. Beijnen, I. Jedema, M. van Zon, I. Mansfield Noringriis, R. Kessels, S. Wilgenhof, H.V. van Thienen, F. Lalezari, A.C.J. van Akkooi, M. Donia, I. Svane. LBA3 - Treatment with tumor-infiltrating lymphocytes (TIL) versus ipilimumab for advanced melanoma: Results from a multicenter, randomized phase III trial. 2022.

[5] Sterner RC, Sterner RM (2021). CAR-T cell therapy: current limitations and potential strategies. Blood Cancer J.

[6] Del Bufalo F, De Angelis B, Caruana I, Del Baldo G, De Ioris MA, Serra A (2023). GD2-CART01 for relapsed or refractory high-risk neuroblastoma. N Engl J Med.

[7] Rosenberg SA, Restifo NP (2015). Adoptive cell transfer as personalized immunotherapy for human cancer. Science.

[8] Kirkin AF, Dzhandzhugazyan KN, Guldberg P, Fang JJ, Andersen RS, Dahl C (2018). Adoptive cancer immunotherapy using DNA-demethylated T helper cells as antigen-presenting cells. Nat Commun.

[9] Werlenius K, Stragliotto G, Strandeus M, Blomstrand M, Caren H, Jakola AS (2021). A randomized phase II trial of efficacy and safety of the immunotherapy ALECSAT as an adjunct to radiotherapy and temozolomide for newly diagnosed glioblastoma. Neurooncol Adv.

[10] Terp MG, Olesen KA, Arnspang EC, Lund RR, Lagerholm BC, Ditzel HJ (2013). Anti-human CD73 monoclonal antibody inhibits metastasis formation in human breast cancer by inducing clustering and internalization of CD73 expressed on the surface of cancer cells. J Immunol.

[11] Gjerstorff MF, Traynor S, Gammelgaard OL, Johansen S, Pedersen CB, Ditzel HJ (2022). PDX Models: a versatile tool for studying the role of myeloid-derived suppressor cells in breast cancer. Cancers (Basel).

[12] Dobin A, Davis CA, Schlesinger F, Drenkow J, Zaleski C, Jha S (2013). STAR: ultrafast universal RNA-seq aligner. Bioinformatics.

[13] Madsen JG, Schmidt SF, Larsen BD, Loft A, Nielsen R, Mandrup S (2015). iRNA-seq: computational method for genome-wide assessment of acute transcriptional regulation from total RNA-seq data. Nucleic Acids Res.

[14] Garbi N, Kreutzberg T (2012). Dendritic cells enhance the antigen sensitivity of T cells. Front Immunol.

[15] Hochweller K, Wabnitz GH, Samstag Y, Suffner J, Hammerling GJ, Garbi N (2010). Dendritic cells control T cell tonic signaling required for responsiveness to foreign antigen. Proc Natl Acad Sci U S A.

[16] Klebanoff CA, Gattinoni L, Palmer DC, Muranski P, Ji Y, Hinrichs CS (2011). Determinants of successful CD8+ T-cell adoptive immunotherapy for large established tumors in mice. Clin Cancer Res.

[17] Radvanyi LG, Bernatchez C, Zhang M, Fox PS, Miller P, Chacon J (2012). Specific lymphocyte subsets predict response to adoptive cell therapy using expanded autologous tumor-infiltrating lymphocytes in metastatic melanoma patients. Clin Cancer Res.

[18] Itzhaki O, Hovav E, Ziporen Y, Levy D, Kubi A, Zikich D (2011). Establishment and large-scale expansion of minimally cultured "young" tumor infiltrating lymphocytes for adoptive transfer therapy. J Immunother.

[19] Mousset CM, Hobo W, Woestenenk R, Preijers F, Dolstra H, van der Waart AB (2019). Comprehensive phenotyping of T cells using flow cytometry. Cytometry A.

[20] Gattinoni L, Klebanoff CA, Palmer DC, Wrzesinski C, Kerstann K, Yu Z (2005). Acquisition of full effector function in vitro paradoxically impairs the in vivo antitumor efficacy of adoptively transferred CD8+ T cells. J Clin Invest.

[21] Schmid P, Chui SY, Emens LA (2019). Atezolizumab and nab-paclitaxel in advanced triple-negative breast cancer. Reply N Engl J Med.

[22] Miles D, Gligorov J, Andre F, Cameron D, Schneeweiss A, Barrios C (2021). Primary results from IMpassion131, a double-blind, placebo-controlled, randomised phase III trial of first-line paclitaxel with or without atezolizumab for unresectable locally advanced/metastatic triple-negative breast cancer. Ann Oncol.

[23] Gammelgaard OL, Terp MG, Preiss B, Ditzel HJ (2018). Human cancer evolution in the context of a human immune system in mice. Mol Oncol.

[24] Gammelgaard OL, Terp MG, Renn C, Labrijn AF, Hamaker O, Nielsen AY (2022). Targeting two distinct epitopes on human CD73 with a bispecific antibody improves anticancer activity. J Immunother Cancer.

[25] Haslam A, Prasad V (2019). Estimation of the Percentage of US patients with cancer who are eligible for and respond to checkpoint inhibitor immunotherapy drugs. JAMA Netw Open.

[26] Loi S, Sirtaine N, Piette F, Salgado R, Viale G, Van Eenoo F (2013). Prognostic and predictive value of tumor-infiltrating lymphocytes in a phase III randomized adjuvant breast cancer trial in node-positive breast cancer comparing the addition of docetaxel to doxorubicin with doxorubicin-based chemotherapy: BIG 02–98. J Clin Oncol.

[27] Loi S, Michiels S, Salgado R, Sirtaine N, Jose V, Fumagalli D (2014). Tumor infiltrating lymphocytes are prognostic in triple negative breast cancer and predictive for trastuzumab benefit in early breast cancer: results from the FinHER trial. Ann Oncol.

[28] Adams S, Gray RJ, Demaria S, Goldstein L, Perez EA, Shulman LN (2014). Prognostic value of tumor-infiltrating lymphocytes in triple-negative breast cancers from two phase III randomized adjuvant breast cancer trials: ECOG 2197 and ECOG 1199. J Clin Oncol.

[29] Cortes J, Rugo HS, Cescon DW, Im SA, Yusof MM, Gallardo C (2022). Pembrolizumab plus chemotherapy in advanced triple-negative breast cancer. N Engl J Med.

[30] Hanahan D, Weinberg RA (2011). Hallmarks of cancer: the next generation. Cell.

[31] Hinrichs CS, Borman ZA, Cassard L, Gattinoni L, Spolski R, Yu Z (2009). Adoptively transferred effector cells derived from naive rather than central memory CD8+ T cells mediate superior antitumor immunity. Proc Natl Acad Sci U S A.

[32] Hinrichs CS, Borman ZA, Gattinoni L, Yu Z, Burns WR, Huang J (2011). Human effector CD8+ T cells derived from naive rather than memory subsets possess superior traits for adoptive immunotherapy. Blood.

[33] Klebanoff CA, Gattinoni L, Torabi-Parizi P, Kerstann K, Cardones AR, Finkelstein SE (2005). Central memory self/tumor-reactive CD8+ T cells confer superior antitumor immunity compared with effector memory T cells. Proc Natl Acad Sci U S A.

[34] Klebanoff CA, Finkelstein SE, Surman DR, Lichtman MK, Gattinoni L, Theoret MR (2004). IL-15 enhances the in vivo antitumor activity of tumor-reactive CD8+ T cells. Proc Natl Acad Sci U S A.

[35] Berger C, Jensen MC, Lansdorp PM, Gough M, Elliott C, Riddell SR (2008). Adoptive transfer of effector CD8+ T cells derived from central memory cells establishes persistent T cell memory in primates. J Clin Invest.

[36] Wang X, Berger C, Wong CW, Forman SJ, Riddell SR, Jensen MC (2011). Engraftment of human central memory-derived effector CD8+ T cells in immunodeficient mice. Blood.

[37] Klebanoff CA, Gattinoni L, Restifo NP (2012). Sorting through subsets: which T-cell populations mediate highly effective adoptive immunotherapy?. J Immunother.

[38] Rosenberg SA, Yang JC, Sherry RM, Kammula US, Hughes MS, Phan GQ (2011). Durable complete responses in heavily pretreated patients with metastatic melanoma using T-cell transfer immunotherapy. Clin Cancer Res.

[39] Hendriks J, Xiao Y, Borst J (2003). CD27 promotes survival of activated T cells and complements CD28 in generation and establishment of the effector T cell pool. J Exp Med.

[40] Buchholz VR, Flossdorf M, Hensel I, Kretschmer L, Weissbrich B, Graf P (2013). Disparate individual fates compose robust CD8+ T cell immunity. Science.

[41] De Togni P, Goellner J, Ruddle NH, Streeter PR, Fick A, Mariathasan S (1994). Abnormal development of peripheral lymphoid organs in mice deficient in lymphotoxin. Science.

[42] Economopoulos V, Noad JC, Krishnamoorthy S, Rutt BK, Foster PJ (2011). Comparing the MRI appearance of the lymph nodes and spleen in wild-type and immuno-deficient mouse strains. PLoS ONE.

[43] Manz MG (2007). Human-hemato-lymphoid-system mice: opportunities and challenges. Immunity.

[44] Taube JM, Anders RA, Young GD, Xu H, Sharma R, McMiller TL (2012). Colocalization of inflammatory response with B7–h1 expression in human melanocytic lesions supports an adaptive resistance mechanism of immune escape. Sci Transl Med.

