# Supplementary figures and images for: Adoptive cell transfer therapy with ex vivo primed peripheral lymphocytes in combination with anti-PDL1 therapy effectively inhibits triple-negative breast cancer growth and metastasis
Source: Mol Cancer. 2024 Jan 6;23:6. doi: 10.1186/s12943-023-01914-8 (PMC10770996; doi:10.1186/s12943-023-01914-8)

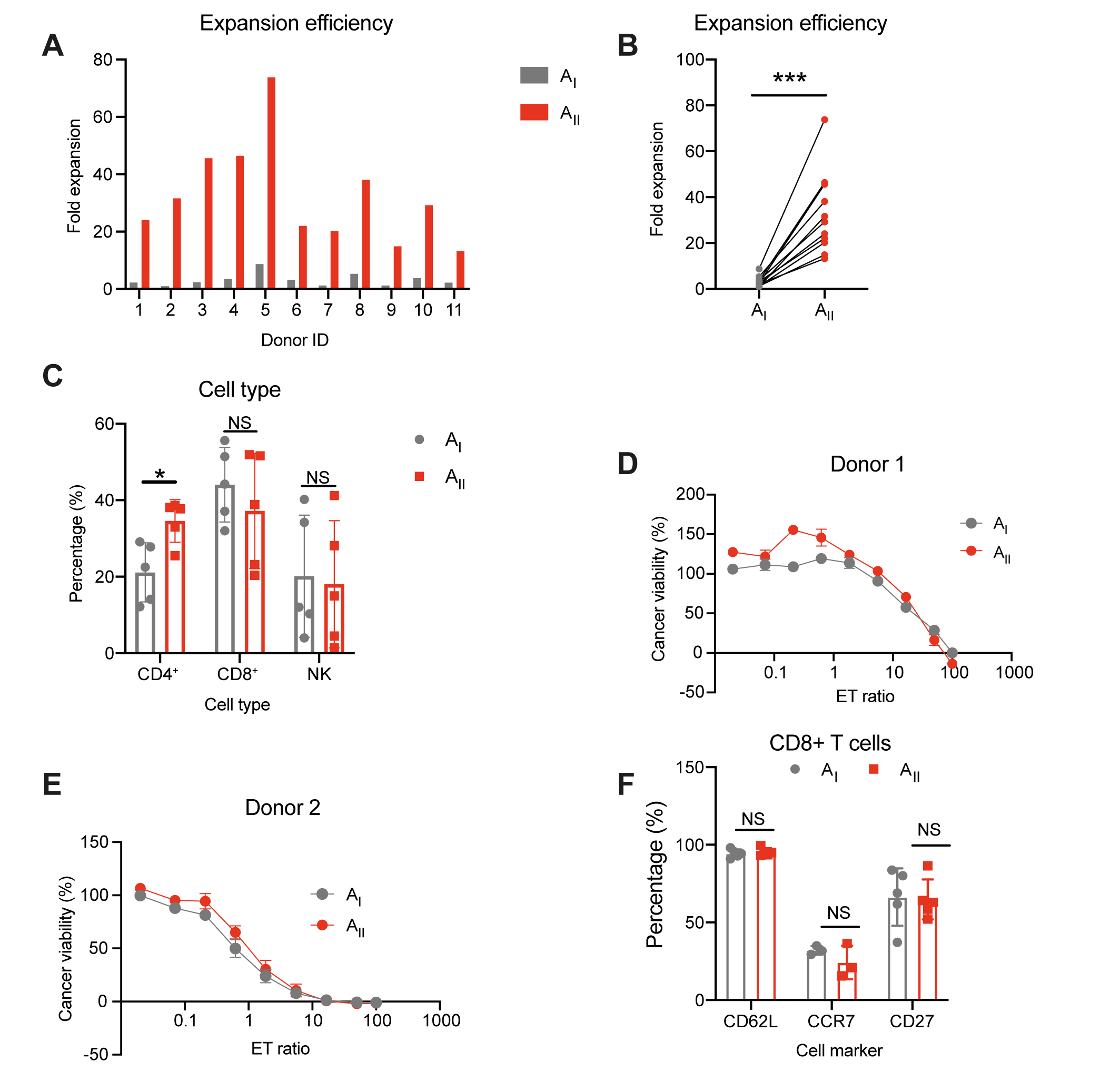

Supplement: Supplementary file 1 — Additional file 1: Supplementary Fig. 1. The ALECSAT II expansion protocol generates a higher number of qualitatively comparable effector cells than the ALECSAT I expansion protocol. A-B Comparison of the total number of generated ALECSAT cells using either the ALECSAT I or II expansion protocols, demonstrating a significant increase using the latter. C Comparison of the proportion of CD4+ or CD8+ T cells as well as NK cells in generated AI and AII products from five different donors, showing a slight increase in CD4+ T cells. D-E Cancer cell viability analysis (luminescence) following 24-hour co-culturing with AI or AII cells generated in parallel from the same donors. Data is presented as mean ± SEM of triplicates. F Phenotypic analysis of CD8+ T cells with regard to CD62L (n=5), CD27 (n=5) and CCR7 (n=3) expression in AI and AII cells generated in parallel determined by flow cytometry. Statistical difference was determined by the paired t-test B or Student’s t-test C and F. *0.05 > P ≥ 0.01, ***0.001 > P [file 12943_2023_1914_MOESM1_ESM.tif]

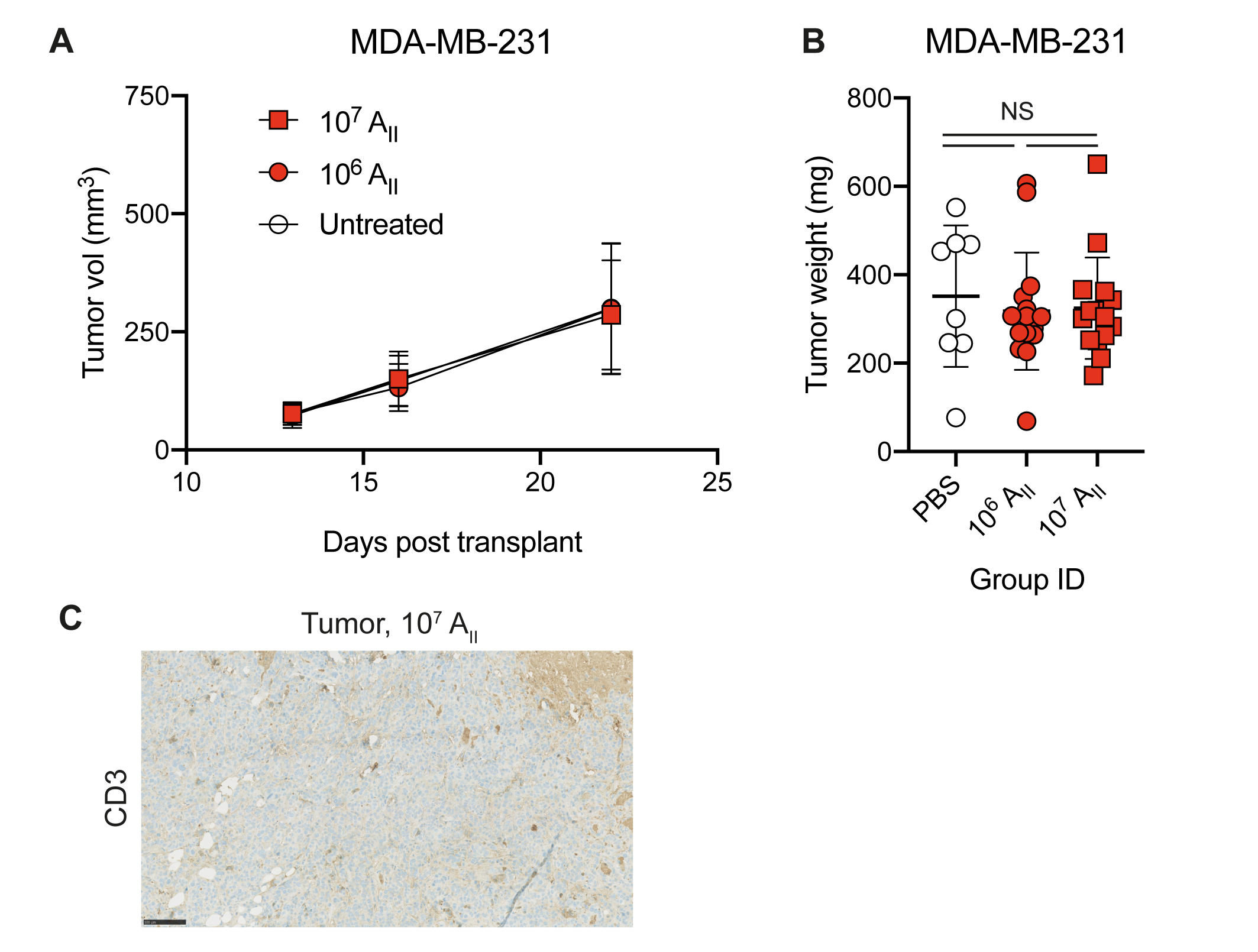

Supplement: Supplementary file 2 — Additional file 2: Supplementary Fig. 2. AII cells perish upon i.v. injection in NOG mice. A Growth of orthotopically transplanted MDA-MB-231 tumors in female NOG mice left untreated (n=8), treated with an i.v. injection of 106 AII cells (n=15) or 107 AII cells (n=15) on day 14. Tumor size was measured on day 13 and mice were randomized to treatment groups. A pool of two AII donors is shown. Data is presented as mean ± SD. B On day 24, tumors from A were excised and tumor mass determined. Data is presented as mean ± SD. C IHC analysis of excised tumors from A and B showing lack of CD3+ cells. Scale bar 100 μm. Statistical difference was determined by Students t-test B. [file 12943_2023_1914_MOESM2_ESM.tif]

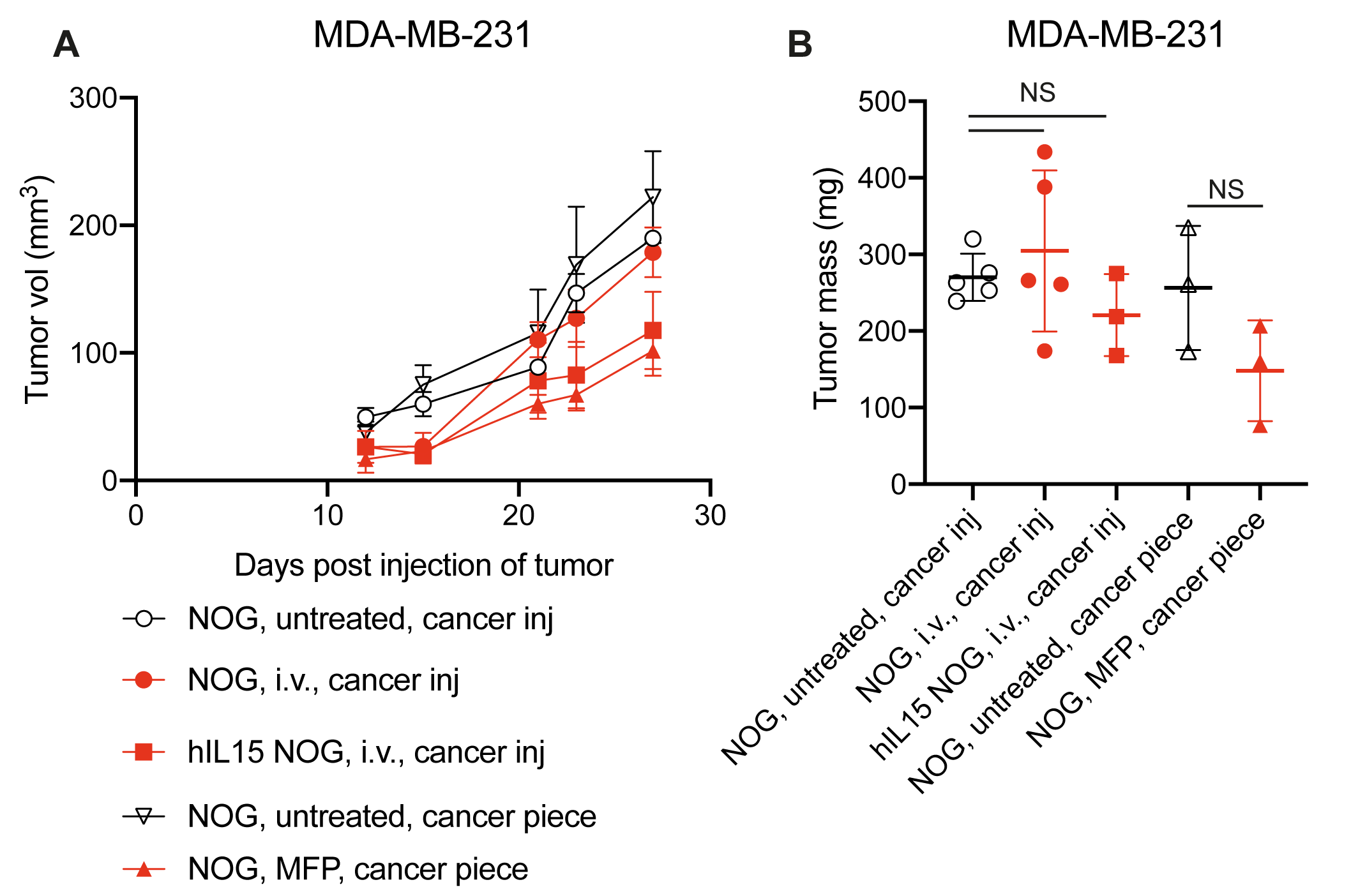

Supplement: Supplementary file 3 — Additional file 3: Supplementary Fig. 3. hIL15 stimulation or injection of AII cells in the vicinity of tumors is insufficient to inhibit tumor growth. A Growth of MDA-MB-231 tumors in female NOG and hIL15 NOG mice. Cancer cells were either injected in suspension or small tumor pieces were transferred into the MFP as indicated in the figure legend. Mice challenged with MDA-MB-231 cells in suspension were either left untreated (n=5) or treated with an i.v. injection of 107 AII cells on day 3 and 9 (n=5, NOG and n=3 hIL15 NOG). Mice receiving MDA-MB-231 tumor pieces were either left untreated (n=3) or treated with an injection of 107 AII directly into the same MFP as the tumor piece on day 0 and an i.v. injection of 107 AII cells on day 9 (n=3). Tumor size is presented as mean ± SEM. B MDA-MB-231 tumors were excised on day 29 and tumor mass were measured and presented as mean ± SD. Statistical difference was determined by the Mann Whitney test B. [file 12943_2023_1914_MOESM3_ESM.tif]

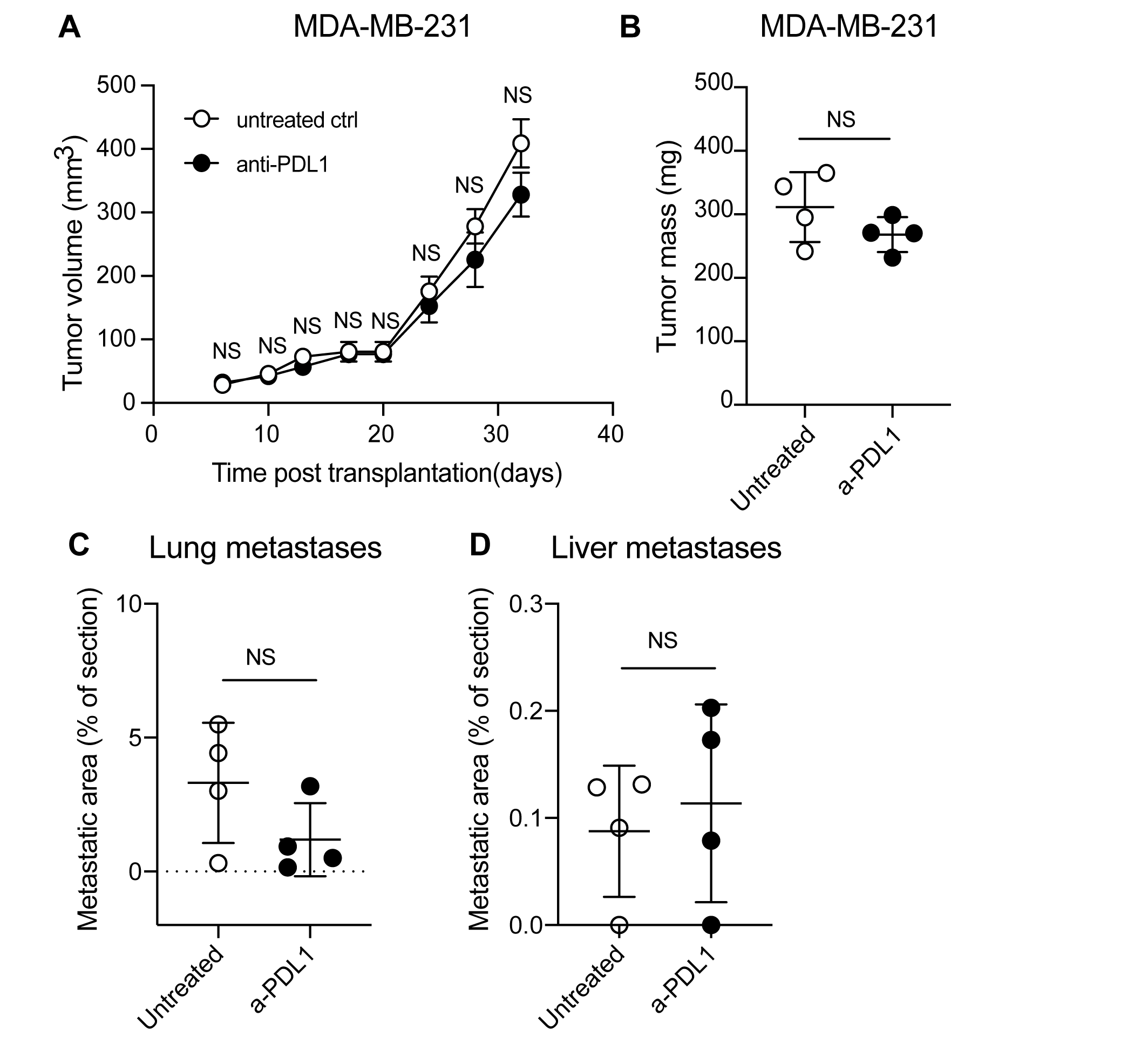

Supplement: Supplementary file 4 — Additional file 4: Supplementary Fig. 4. Anti-PDL1 therapy is ineffective as monotherapy in NOG mice. A Growth of orthotopically-transplanted MDA-MB-231 tumors in female NOG mice left untreated (n=4) or treated with anti-PDL1 (n=4). (B) On day 33, tumors from A were excised and tumor mass determined. Data is presented as mean ± SD. Quantification of spontaneous lung C and liver D metastases from A presented as mean ± SD. Statistical differences were determined by the two-way ANOVA method following Bonferoni’s multiple correction testing A or the Student’s t-test B-D, respectively *0.05 > P ≥ 0.01, **0.01 > P ≥ 0.001, ***0.001 > P. a-PDL1, anti-PDL1. [file 12943_2023_1914_MOESM4_ESM.tif]

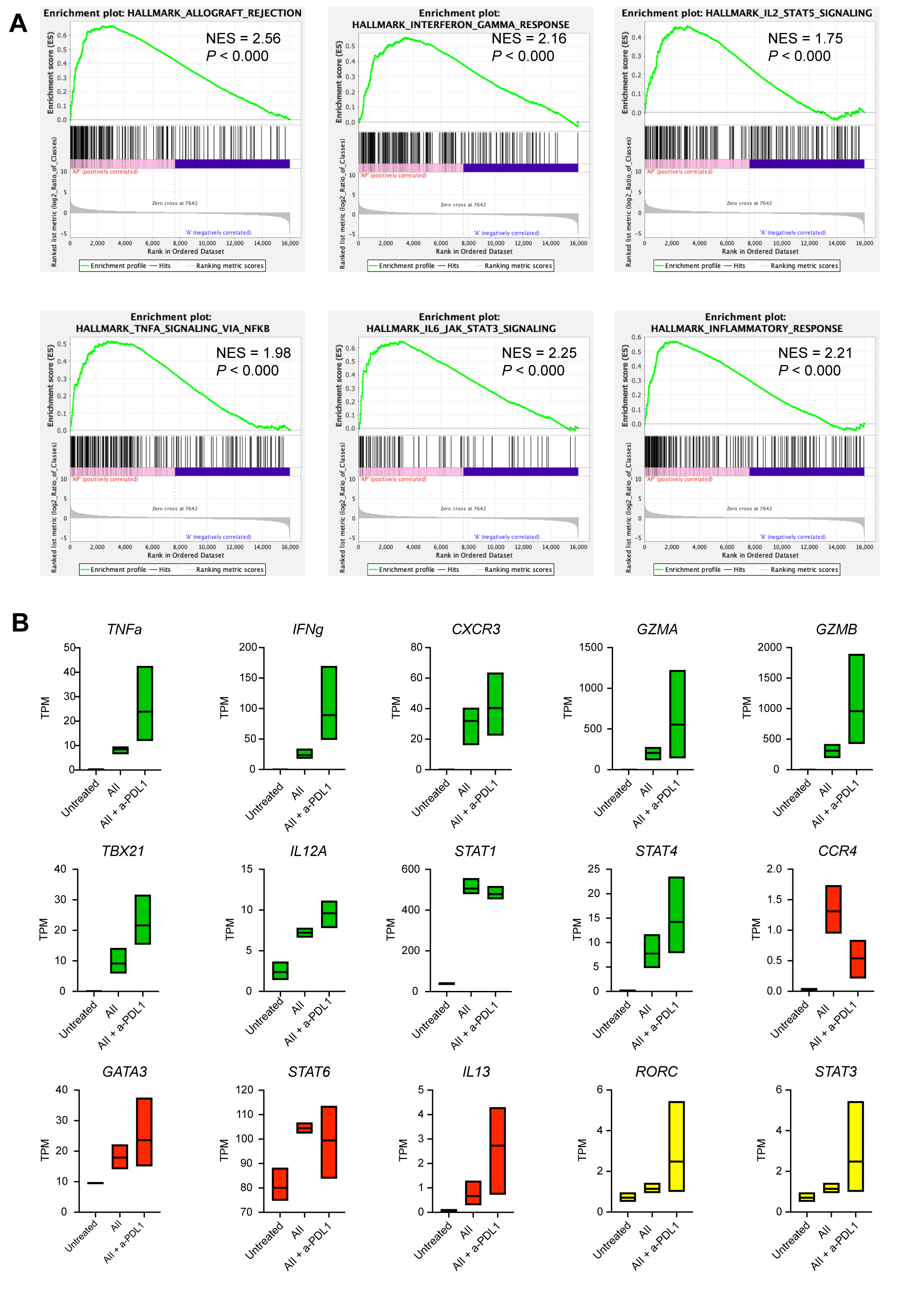

Supplement: Supplementary file 5 — Additional file 5: Supplementary Fig. 5. Cancer control is associated with T cell activity. A Enrichment plots of significantly enriched gene sets in tumors treated with AII in combination with anti-PDL1 compared to those treated with AII as monotherapy showing strengthened T cell responses. B Comparison of selected genes associated with Th1 (green), Th2 (red) and Th17 responses (yellow) showing a consistent increase in Th1-, but not Th2- or Th17-associated genes. [file 12943_2023_1914_MOESM5_ESM.tif]

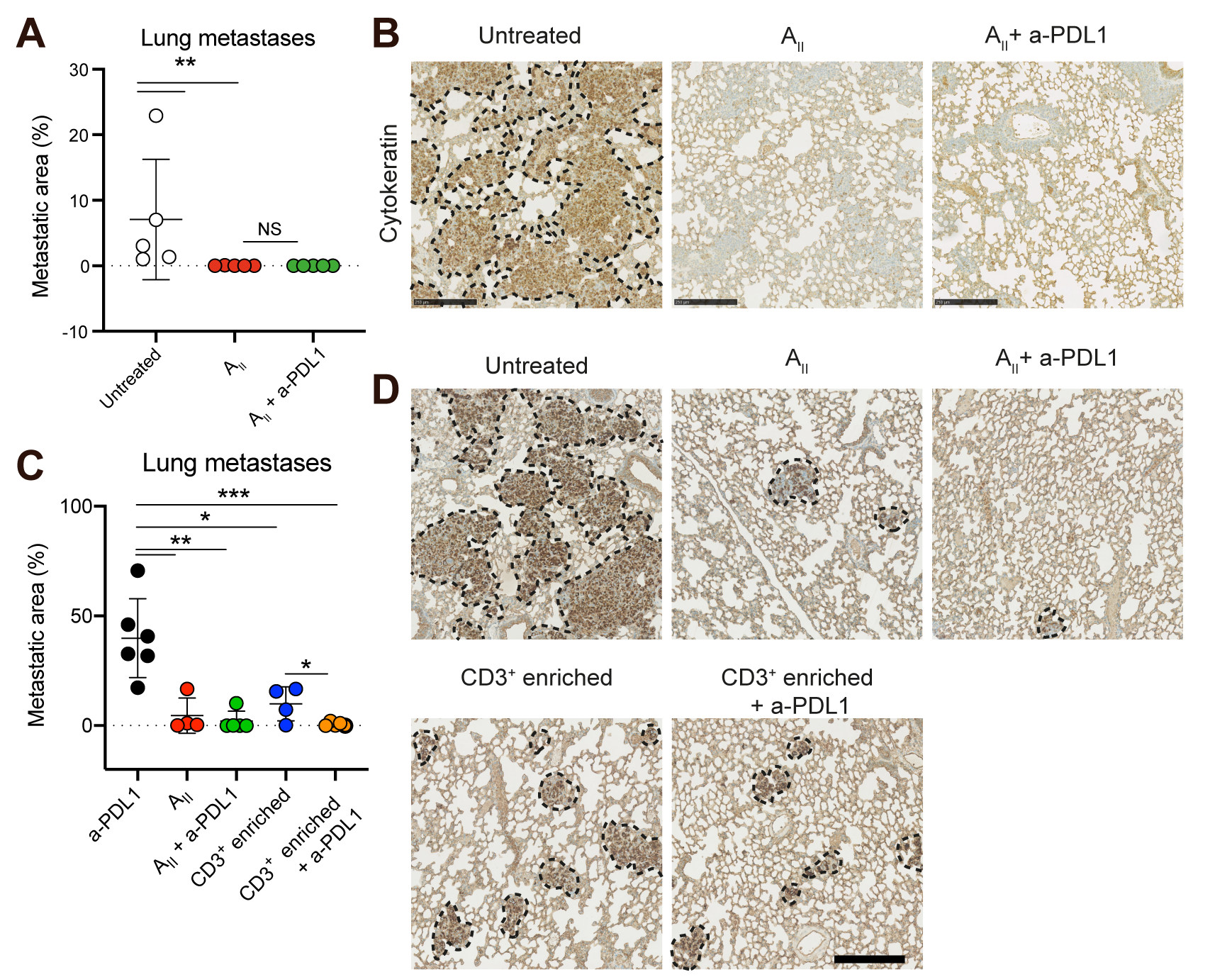

Supplement: Supplementary file 6 — Additional file 6: Supplementary Fig. 6. AII cells suppress spontaneous metastasis formation in NOG mice. A Quantification of spontaneous lung metastases from the animals presented in figure 3e presented as mean ± SD. B Representative IHC panels of lungs stained for pan-cytokeratin from A. Dotted lines represent tumor borders. C-D As in A-B with primary tumor expansion shown in figure 3a. Statistical difference was determined by the Mann Whitney A or unpaired t-test C, respectively *0.05 > P ≥ 0.01, **0.01 > P ≥ 0.001, ***0.001 > P. NS, non-significant; a-PDL1, anti-PDL1. Black scale bar 250. [file 12943_2023_1914_MOESM6_ESM.tif]
